# The use of head helmets to deliver noninvasive ventilatory support: a comprehensive review of technical aspects and clinical findings

**DOI:** 10.1186/s13054-021-03746-8

**Published:** 2021-09-08

**Authors:** Andrea Coppadoro, Elisabetta Zago, Fabio Pavan, Giuseppe Foti, Giacomo Bellani

**Affiliations:** 1 ASST Monza, San Gerardo Hospital, Monza, Italy; 2 Department of Medicine and Surgery, University of Milan-Bicocca, Via Cadore 48, Monza, MB Italy

**Keywords:** Noninvasive ventilation, Helmets, COVID-19, Acute respiratory distress syndrome, Continuous positive airway pressure

## Abstract

A helmet, comprising a transparent hood and a soft collar, surrounding the patient’s head can be used to deliver noninvasive ventilatory support, both as continuous positive airway pressure and noninvasive positive pressure ventilation (NPPV), the latter providing active support for inspiration. In this review, we summarize the technical aspects relevant to this device, particularly how to prevent CO_2_ rebreathing and improve patient–ventilator synchrony during NPPV. Clinical studies describe the application of helmets in cardiogenic pulmonary oedema, pneumonia, COVID-19, postextubation and immune suppression. A section is dedicated to paediatric use. In summary, helmet therapy can be used safely and effectively to provide NIV during hypoxemic respiratory failure, improving oxygenation and possibly leading to better patient-centred outcomes than other interfaces.

**Supplementary Information:**

The online version contains supplementary material available at 10.1186/s13054-021-03746-8.

## Introduction

Noninvasive ventilatory support (NIV) is frequently used in the treatment of several forms of acute (or acute-on-chronic) respiratory failure. During the COVID-19 pandemic, increased attention has been devoted to the use of helmets. Helmets have been in use since the early 2000s [1–3], albeit mostly only in a few countries, Italy in particular [4]. Given the increasing use of this interface, we considered it worth summarizing the available knowledge on the topic.

A helmet is constituted by a soft (but nonextensible) transparent hood that fits over the patient’s entire head without any contact point and is anchored (in some cases by a rigid ring) to a soft and extensible collar that fits gently around the patient’s neck. The helmet typically has two (or more) connectors for the gas inlet and outlet; O_2_-enriched gas can be provided by a Venturi system, a turbine flow generator or a ventilator. As discussed in detail below, the advantages of helmets result from their tolerability (noise representing a possible limitation), cost-effectiveness and excellent sealing capability (minimizing leaks), the latter being obtained easily and involving very gentle contact, resulting in minimal risk of soft tissue injury. This review is divided into two main sections. The first is dedicated to the use of helmets in the delivery of continuous positive airway pressure (H-CPAP), typically powered by a continuous free-flow system and a PEEP valve. During CPAP, the patient is free to inhale or exhale, while the pressure within the helmet remains constant, and there is no interaction with a ventilator and no “active" inspiratory support. The second section is dedicated to noninvasive positive pressure ventilation (NPPV), which offers active support for inspiration (typically by pressure support) delivered by a mechanical ventilator. CPAP and NPPV are often lumped together under the broad umbrella of “NIV”, but distinguishing between these two forms of support is crucial. Particularly in the context of helmets, CPAP and NPPV offer two completely different approaches and mechanisms of action; for reader convenience, we consider paediatric use separately, but the considerations discussed above still apply.

## Methods

We searched PubMed for records published until April 30, 2021, using the following keywords: “helmet CPAP”, “CPAP noninvasive ventilation”, “helmet ventilation”, “helmet pressure support” and “helmet COVID-19” for a total of 559 screened records.

We included articles published in the English language only. Additional file 1 contains a list of excluded articles because they were not relevant (e.g. motorcycle helmets) or because they were reviews, editorial articles, case reports or series with fewer than ten cases. Eventually, 112 studies were identified and included in this review.

## Use of helmets to deliver CPAP

### Technical principles of H-CPAP

As outlined below, the main advantage of delivering CPAP by helmet instead of by face mask is that it offers better pneumatic performance with free-flow systems and is associated with greater patient tolerance; the greatest drawback is the risk of possible CO_2_ rebreathing.

**Fig. 1:**
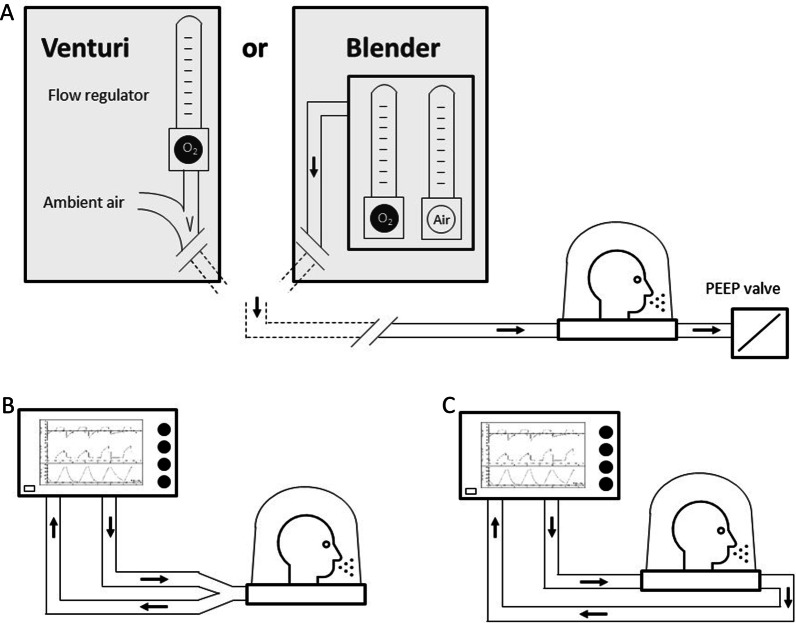
Schematic drawings of the main helmet circuit configuration possibilities. For free-flow continuous positive airway pressure (CPAP, **A**), the gas mixture may be generated with either a Venturi system empowered by an oxygen source or an oxygen/air blender. The gas mixture flows through the helmet and is dispersed through a PEEP valve, which maintains a constant positive pressure backwards. An alternative configuration involves the connection of the helmet with a mechanical ventilator to provide noninvasive positive pressure ventilation, typically with the pressure support mode (NPPV) by either a single port (**B**) connected to the circuit Y piece (condition associated with a higher risk of CO_2_ rebreathing, see text) or two separate ports (**C**)

The simplest configuration of H-CPAP involves a constant flow of fresh gas (at variable FiO_2_) through the helmet that is dispersed in ambient air through a positive end-expiratory pressure (PEEP) valve connected to the expiratory helmet port (Fig. 1, Additional file 2: Figure e1).

An adequate flow of fresh gas in the helmet [5] is required for two main purposes: keeping the positive pressure by passing through the expiratory valve and preventing CO_2_ rebreathing. In regard to the first aspect, it is worth noting that H-CPAP requires lower fresh gas flows (in the range of 60 l/min) than face masks (flows up to 100 or 120 l/min) [6]. With a helmet, the airway pressure is also stable if the patient’s peak inspiratory flow exceeds the fresh gas flow because of its high compliance (i.e. internal volume variations are accommodated with low-pressure variations). Conversely, the pressure in a “rigid” (i.e. with low compliance) system, such as a face mask, drops when the patient’s peak demand is higher than the gas flow, resulting in additional work for the patient and possibly reduced alveolar end-expiratory pressure.

When using a helmet, assuring adequate washout of CO_2_ is of paramount importance. Patroniti et al. [7] showed that a fresh gas flow rate below 40 l/min leads to significant rebreathing of CO_2_ during inspiration. In line with these findings, Taccone et al. confirmed that significant CO_2_ rebreathing is present with a fresh gas flow rate of 30 l/min [8]. The same paper showed that the use of a mechanical ventilator, set in CPAP mode, should be absolutely avoided with a helmet: in this condition, the circulation of gas flowing through the system is similar to the patient’s minute ventilation and hence totally inadequate to wash CO_2_ [8].

Different helmet brands and models vary in terms of sizing and the presence of auxiliary inputs and anchoring systems. Some helmets are equipped with anti-suffocation valves, which allow the patient to breathe room air in the case of fresh gas failure supply [9, 10]. Some authors tested an interface combining high-flow nasal cannulas and H-CPAP in healthy volunteers [11].

On the expiratory limb, it is possible to employ either a water sealed or (more practically) mechanical valve. The ideal valve employs a threshold, rather than a constant, resistance, so that the pressure within the helmet remains constant irrespective of the flow [12].

The possibility of alternating two different PEEP valves on the expiratory limb has also been described as a way to provide nonsynchronized alternating pressure within the helmet, which can improve gas exchange in hypoxemic patients [13, 14].

High-flow nasal oxygen is gaining widespread use: it allows delivery of a known FiO2 and a mild level of PEEP. It is likely that this device might be as effective as H-CPAP, particularly in less severe patients, although a direct comparison is missing.

### Clinical evidence for helmet CPAP

The efficacy of NIV is well known during acute respiratory failure caused by cardiogenic pulmonary oedema (CPE), where NIV reduces the intubation rate and mortality [15].

H-CPAP appears to be an effective alternative to standard facemasks during CPE, even in cases of severe respiratory acidosis and hypercapnia [16]; in one of the earliest clinical studies on helmet use, Tonnelier et al. showed that PaCO_2_ progressively decreases towards normal values during the first 24 hours of H-CPAP treatment. In the same study, H-CPAP was applied in 11 patients and allowing CPAP to be applied for several hours without any reported adverse events or clinical intolerance [17].

H-CPAP in CPE patients is feasible and can be safely applied in the prehospital setting. Foti et al. showed that early H-CPAP led to sudden and sustained improvement in respiratory function (peripheral oxygen saturation increased from 79 ± 12 to 97 ± 3%, and respiratory rate decreased from 26 ± 4 to 21 ± 3 breaths per minute) and circulatory function (systolic blood pressure decreased from 175 ± 49 to 145 ± 28, and heart rate decreased from 112 ± 23 to 105 ± 19). H-CPAP even benefited patients rescued by nursing personnel only, hence in the absence of any pharmacological intervention [18, 19], implying that CPAP should be used as a first-line intervention even before standard medical treatment.

In the context of community-acquired pneumonia (CAP), a randomized controlled trial by Cosentini et al. in 2010 demonstrated how H-CPAP, in comparison with standard oxygen therapy using a Venturi mask, improved oxygenation faster (PaO_2_/FiO_2_ ratio ≥ 315 in 1.5 h vs. 48 h) and in a greater number of patients (95% of patients vs. 30%); however, improvements in oxygenation were lost after discontinuation of CPAP [20].

Moreover, in 2014, Brambilla et al. demonstrated that H-CPAP, compared to standard oxygen, reduced the risk of endotracheal intubation, demonstrating the beneficial effects of this technique on a relevant clinical outcome [21].

H-CPAP can be a safe and effective treatment option for immunocompromised patients with ARF. Rabitsch et al. demonstrated that better tolerance to NIV achieved by using a helmet can lead to a higher rate of successful treatment (defined as an improved PaO_2_/FiO_2_ and decreased PaCO_2_ and respiratory rate) and might improve survival rates in immunocompromised patients [22]. Similarly, in a study from 2004 by Principi et al., CPAP tolerance was higher with helmet than face masks and was associated with a 49% reduction in the risk of death [23].

The feasibility and clinical effectiveness of H-CPAP in patients who developed ARF following surgery were demonstrated by several studies, in which H-CPAP was associated with improved gaseous exchange, preventing hypoxemia development and the need for endotracheal intubation [24–26].

Prophylactic postoperative H-CPAP in nonhypoxemic patients following pulmonary lobectomy transiently improved oxygenation and was associated with shorter hospital stays [27].

### COVID-19 experience

Patients with coronavirus disease 2019 (COVID-19) pneumonia can develop severe hypoxemia and require PEEP, although these patients are at greater risk of NIV failure than patients with ARF with other aetiologies [28]. Some authors have suggested that helmets might reduce aerosol spread [29, 30], as confirmed by some exploratory studies [31, 32].

Several authors have reported on the use of H-CPAP inside [33, 34] and outside the ICU in COVID-19 resource shortages [35–38]. Among others, Bellani et al. demonstrated, in a single-day observational study, that the H-CPAP success rate was greater than 60%, and close to 75%, in patients without a do-not-intubate (DNI) order. This study also highlighted known factors independently associated with NIV failure, such as age and PaO_2_/FiO_2_ (threshold value of 150 mmHg), and others more specific to COVID-19, such as serum levels of C-reactive protein and platelet counts. [39].

**Fig. 2:**
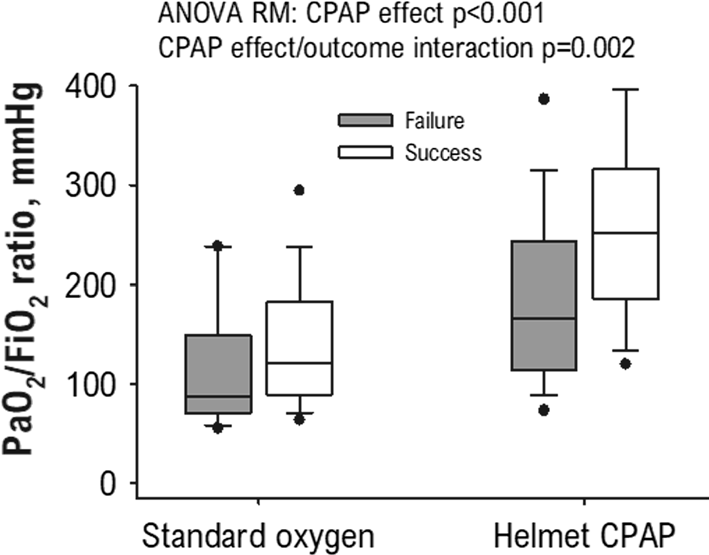
Helmet CPAP therapy markedly improves oxygenation in COVID-19 patients (PaO_2_/FiO_2_ nearly doubles compared to standard oxygen therapy, *P* < 0.001 for CPAP effect at ANOVA RM). The oxygenation increase was more pronounced in patients who could be successfully treated with helmet CPAP without escalation to intubation (white boxes, *P* = 0.002 for interaction between the CPAP effect and outcome). Reproduced under Creative Common licence from [40]

In another study by Coppadoro et al., H-CPAP treatment after standard oxygen therapy failure was feasible for several days outside the ICU, despite persistent impairment in gas exchange. Helmet CPAP treatment was successful in 69% of patients without a DNI order, but DNI patients could also benefit from helmet CPAP as rescue therapy. Successful treatment with H-CPAP (hospital discharge without intubation) was associated with a nearly double response in oxygenation to the therapy (PaO_2_/FiO_2_ ratio increase from 103 to 202 mmHg). In other words, as shown in Fig. 2, positive pressure not only improved oxygenation but also allowed better stratification of patient severity [40]. Some authors also suggested that an incremental PEEP trial might allow a PEEP selection with optimized oxygenation while avoiding haemodynamic complications [41].

Another observational, prospective study by Retucci et al. showed that patients with ARF might also benefit from prone positioning; H-CPAP allows safe early self-proning in awake, spontaneously breathing and nonintubated patients [42].

We found only one study on helmet NPPV in COVID: Grieco et al. compared helmet NPPV to high-flow nasal oxygen (HFNO) showing that, 48 h after randomization, patients treated with helmet NPPV had better oxygenation, a lower respiratory rate and lower hypocapnia, albeit with greater device-related discomfort. The primary endpoint (days free of respiratory support) did not differ between the two arms, but the intubation rate was lower in patients treated with helmet therapy than in those treated with HFNO (30% vs. 51%, *P* < 0.03) [43].

## Use of helmets to deliver NPPV

The most common method for providing assisted ventilation noninvasively (noninvasive positive pressure ventilation, NPPV) is with the use of a face mask connected to a ventilator. A positive pressure above the set PEEP level is delivered through an interface covering the patient’s airway; helmets have been proposed to replace face masks due to the lower rate of complications during long-term therapy (e.g. pressure ulcers), with a comfort comparable to that of HFNO [44]. Moreover, end-expiratory lung volume is higher during helmet NPPV than during face mask NPPV, possibly due to reduced activation of expiratory muscles [45]. However, adequate pressurization of the large internal volume helmet and patient–ventilator interaction might be difficult to obtain: pressure support ventilation (PSV) is more efficiently delivered by a face mask than a helmet in terms of reduced work of breathing, lower time to reach the target pressure and higher airway pressure–time product during PSV [5]. Moreover, PSV delivered by a helmet is less effective in removing CO_2_ and is associated with a higher number of asynchronies than PSV delivered by a face mask [46]. The tidal volume measurement provided by the ventilator is not reliable when ventilating through a helmet, although recent reports suggest that such a measurement might be feasible with dedicated equipment [47].

### Use of specific ventilator settings

In healthy subjects, increasing the level of pressure support during helmet NPPV results in increased tidal volume and reduced respiratory efforts [48]. However, the large helmet inner volume and compliance lead to delayed pressurization and reduced inspiratory pressure in the patient’s airways, resulting in impaired patient–ventilator synchrony. Therefore, specific ventilator settings should be chosen when delivering NPPV thorough a helmet, such as a higher PEEP to stiffen the helmet, increased PSV level, higher pressurization time (i.e. low rise time) and cycling-off flow threshold [49–51].

To overcome the issues of slow pressurization and patient–ventilation interaction, novel helmets have been designed specifically for NPPV: a smaller internal volume and lower compliance resulted in better interaction in a bench study [52], while to improve comfort and synchrony, innovative helmet designs involve an internal inflatable collar [53, 54].

In healthy subjects undergoing NPPV, even an optimized helmet was not as efficient as a face mask with respect to ventilator triggering and cycling at low PEEP and PSV levels; at higher levels, it performed similarly to the face mask, with the advantage of reduced inspiratory effort [55]. The advantages of the novel helmet compared to the standard one were confirmed in a cohort of postextubation patients [56].

**Fig. 3:**
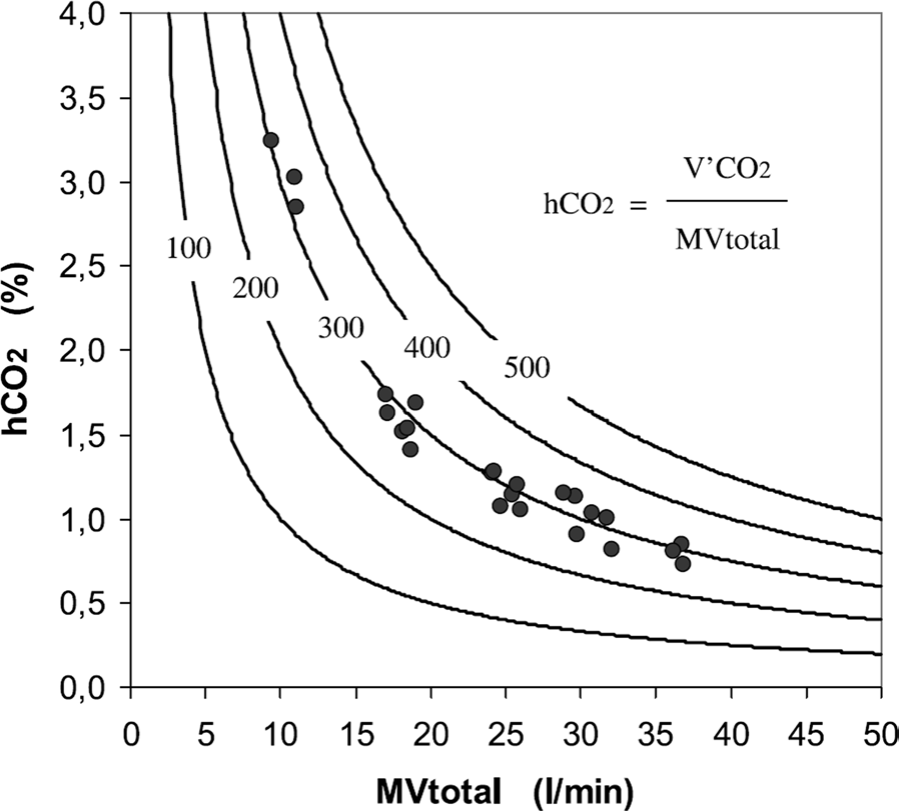
The amount of "fresh" gas flowing through the helmet (MVtotal) determines the average CO_2_ concentration within the helmet (hCO_2_). Circles represent measured data, while lines are the theoretical curves obtained by the equation reported in the graph at different levels of CO_2_ production. Reproduced from [59]

CO_2_ rebreathing is a key issue during helmet NPPV due to the greater amount of dead space than in a face mask; however, the effective dead space might be less than expected, as shown by mathematical modelling [57, 58]. The average helmet CO_2_ concentration depends primarily on CO_2_ production and total helmet ventilation (monitored by the ventilator as “minute ventilation”): higher pressure support levels, leading to increased minute ventilation, result in better CO_2_ washout (Fig. 3) [59].

The average helmet CO_2_ concentration can be quite high, reaching 18 mmHg when using a standard double-limb ventilator circuit connected to the helmet through a y-piece (Fig. 1); if the ventilator provides a flow-by and the circuit limbs are connected to two independent helmet ports without the y-piece, the helmet CO_2_ concentration is halved.

Compared to a standard double-limb circuit connected to the inspiratory and expiratory ports of the helmet, a single limb circuit with a modified expiratory valve placed on the helmet’s expiratory port (open circuit) provides better CO_2_ washout (PiCO_2_ reduced from 10 to 5 mmHg) but with slower pressurization [60].

Compared with PSV, nonsynchronized high-flow biphasic positive airway pressure allows more efficient CO_2_ removal but with much worse patient–ventilator interaction [61].

### Patient–ventilator interaction

Helmet NPPV is burdened by longer trigger delays and associated with increased tidal volumes, but asynchronies might be difficult to identify from ventilator traces [62, 63]. The use of a double-limb circuit connected to the inspiratory and expiratory ports (no y-piece) improved synchrony in a bench study [64]. The cycling-off threshold (switch from inspiration to expiration) should be maintained at high levels (> 30%), particularly in COPD patients, as demonstrated in another bench study [65]. To obtain the best coupling between the neural inspiratory effort and pressure delivery by the ventilator, the use of assisted ventilation based on diaphragm electrical activity was investigated. In healthy subjects, patient–ventilator synchrony was improved, particularly at higher levels of support and respiratory rates [65, 66]. A better patient–ventilator interaction was also confirmed in two cohorts of postextubation patients [67, 68].

### Clinical indications and feasibility of helmet NPPV

#### Acute hypoxemic respiratory failure

In patients with hypoxemic respiratory failure treated by PSV, helmets were effective, and tolerance was higher than it was for face masks [69, 70]. Among hypoxemic patients affected by community-acquired pneumonia, approximately 40% of the enrolled subjects were successfully treated with helmet NPPV; poor PaO_2_/FiO_2_ improvement after beginning helmet NPPV was associated with helmet failure and a subsequent need for intubation [71]. Despite sporadic use of the helmet in the USA, in 2016, Patel et al. conducted a pivotal randomized clinical trial on 200 patients comparing NPPV delivered by helmet vs. face mask in ARDS patients; the helmet proved superior, leading to a reduced need for intubation, lower ICU-acquired weakness and lower mortality rates [72]. The superiority of helmets is even more remarkable considering that the ventilator setting with the helmet was not optimized according to the principles described above and the likelihood that some CO_2_ rebreathing occurred. Moreover, helmet use could also be cost-effective [73, 74]. Helmet NPPV might also be used as an alternative to invasive ventilation during the weaning phase, with a similar ventilatory support duration but fewer infectious complications [75].

#### COPD exacerbation

Helmet NPPV was feasible for treatment of COPD exacerbation, although it was inferior to face mask NPPV for CO_2_ removal in an observational trial [26]. Two randomized trials confirmed that face mask ventilation is associated with significant PaCO_2_ reductions in COPD patients and that helmet NPPV is not comparably efficient [76, 77].

In a crossover clinical trial on a small population of patients with COPD, helmets and face masks were comparably tolerated and effective in improving hypercapnia; however, lower inspiratory efforts and better patient/ventilator interactions were recorded with face masks [78].

A later randomized clinical trial enrolling 80 patients with COPD exacerbation confirmed the same findings: helmets were equivalent to face masks in terms of discomfort, blood gas improvement and rate of intubation, while dyspnoea was reduced more effectively by face masks [79].

To overcome the synchronization and pressurization issues related to helmet NPPV, a system based on neurally adjusted ventilatory assist for helmet NPPV was tested in a small cohort of COPD patients, resulting in improved comfort and similar respiratory patterns and breathing efforts compared to face masks [80].

#### Other populations

Helmet NPPV was also feasible in hypoxemic immunocompromised patients, leading to better patient tolerance, fewer skin complications and lower discontinuation rates than face masks [81]. The use of helmet NPPV in postoperative respiratory failure patients was associated with a lower need for intubation and better tolerance [82].

#### Key points for helmet NPPV

Issues related to helmet NPPV are slow helmet pressurization, reduced CO_2_ washout and patient–ventilator asynchrony. Helmet NPPV is superior to face mask NPPV in ARDS patients and can be successfully used to treat hypoxemic patients; however, helmet NPPV is inferior in COPD patients. For optimal NPPV delivery, one should consider (1) helmets specifically designed for NPPV; (2) proper ventilator circuit connections to helmet inlet and outlet ports, avoiding the use of filters; (3) specific ventilator settings (high PEEP and assistance levels, low rise time and early cycling to expiration); and (4) neurally coupled ventilation to improve synchrony, particularly in COPD patients.

## Patient comfort, complications and other practical issues

Patient comfort is essential during NIV to reduce potential complications leading to endotracheal intubation. Here, we summarize some practical interventions devised mainly for H-CPAP that are likely applicable to NPPV.

Some authors suggested that low-dose remifentanil could increase patients’ tolerance to helmet and face mask NPPV [83]. Lucchini et al. proposed a “bundle of interventions” aimed at increasing the comfort of patients treated with helmet CPAP to increase the duration of treatment, including noise reduction [29].

The WHO guidelines recommend limiting ICU noise levels to between 45 and 60 dB during the daytime and 35 dB during the night-time. When a Venturi system is used to generate flow, the noise exposure is significantly more intense than ICU noise [84, 85], which may increase patient discomfort and affect ear function [86]; moreover, noise may decrease the acceptance of helmet use during long-term treatments.

Noise exposure during H-CPAP may be attenuated by positioning heat-moisture exchange filters on the inspiratory limb [87]. Other tools proposed to decrease patients’ perceived noise include earplugs, sound traps and tubes with smooth inner surfaces [84, 85] and avoiding unnecessarily high flows.

Particularly, when dry medical gas is used for helmet CPAP, gas humidification can be far below the recommended value (10 mg H2O/l) [88].

The use of a heated humidifier allows adequate humidification while avoiding condensation but does not affect patients’ level of comfort [88]; the humidifier should be adjusted at 26 °C with a temperature gradient that increases towards the patient (+ 2 °C) [89]. Others have suggested that the best comfort is obtained by humidifying without heating [90].

The two most commonly used solutions for fixing H-CPAP setups (a relevant aspect affecting patient tolerance) are armpit straps and counterweight systems. The armpit strap option may cause pain and pressure ulcers. The counterweight system seems to minimize these risks, yielding better tolerance (the force of gravity generated plus the placement of a pad cushion reduces the contact between skin and the device.) Delivering helmet CPAP with an armpit strap fixing system should be planned for short periods of time (no more than 2 h). The counterweight option is indicated in the case of prolonged CPAP helmet cycles [91].

Unfortunately, while much attention has been devoted to patient (dis)comfort, only a few studies have systematically assessed and reported other types of complications. Specifically, adverse events (which are also the most relevant ones, in the opinion of the authors), including headache, otalgia, sensation of claustrophobia, cutaneous sores and ulcerations, have seldom been monitored. However, the incidence of these was found to be very low [17, 21, 72].

## Use in the paediatric population

In recent years, NIV has been increasingly applied to paediatric patients with different indications and settings [92]. NIV is also indicated in immunocompromised patients to avoid infectious complications following intubation [92, 93].

One of the key issues when delivering NIV among children is the interface. Among young children, H-CPAP can be used with a device modified in terms of size, with fastening achieved by a device called a "baby-body" [94]. With this kind of fastening system, the helmet is fastened around the baby's bottom instead of the classical armpits [95]. Even if H-CPAP can be as effective as nasal mask prongs to treat mild ARF or apnoea in preterm newborns [96], the latter are still the main interfaces used for newborns. This choice might also be explained by the higher noise of H-CPAP versus nasal prongs [97] and by the easier access to the baby to provide care with nasal mask/prongs. Moreover, H-CPAP seems to reduce cerebral blood flow more than nasal masks [98]. Some authors have suggested that helmets allow effective delivery of nitric oxide [99] or aerosols [100].

Some authors tried to compare H-CPAP to nasal-facial masks among toddlers and children in terms of tolerance, efficacy and feasibility [101, 102]. Specifically, Chidini et al. [103, 104], in a randomized trial, found that H-CPAP had a lower treatment failure rate due to intolerance (3/17 [17%] vs. 7/13 [54%], *P* = 0.009), and fewer infants required sedation (6/17 [35%] vs. 13/13 [100%], *P* = 0.023). Moreover, they showed that H-CPAP is safe, even for prolonged use in acute clinical settings [105].

Since toddlers affected by bronchiolitis can also be treated with HFNO, one recent randomized controlled trial compared the efficacy of H-CPAP with HFNO: both systems were effective in improving the clinical conditions of patients with mild-to-moderate respiratory distress, and the response to helmet CPAP was more pronounced and rapid than that to HFNO, with a shorter hospitalization duration (4.9 vs. 13.1 Days *P* = 0.001) and less use of steroids and salbutamol (3 vs. 7 Days *P* = 0.009) in the first group [106]. The efficacy of H-CPAP in bronchiolitis was also recently reported in a retrospective study [107]. Finally, post-transplant extubation respiratory failure has been effectively treated with H-CPAP [108].

Nasal or facial masks are the primary interfaces used for chronically ventilated patients (neuromuscular or genetic syndromes), with complication rates of up to 21% (discomfort, leaks, skin injuries), mandating systematic and close monitoring of the NPPV interface [109]. The lack of adoption of helmets in this setting can be explained by the difficulties associated with synchronization during pressure support: as reported by Conti et al. in one experimental model, helmets demonstrated the worst interaction (longest inspiratory trigger delay compared with the endotracheal tube and face mask), suggesting that face masks should be considered the first choice for delivering NPPV in children [110].

## Conclusions

Relevant evidence has been published in the last 20 years, and several trials are ongoing [111–113]. The tragic COVID experience has led to more widespread use of helmets. Different technical solutions can be applied (free-flow CPAP vs. mechanical ventilator NPPV), and no data are available to establish whether either technique is superior. In any case, an adequate fresh gas flow must be provided to avoid CO_2_ rebreathing. As summarized above and by several meta-analyses [114–119], helmet therapy can be safely and effectively used to provide NIV during hypoxemic respiratory failure, better improving oxygenation than standard oxygen mask treatment and possibly leading to better patient-centred outcomes than other NIV interfaces.

## Supplementary Information

**Additional file 1**. List of papers retrived by literatire search but excluded from the review.

**Additional file 2**. Color image of helmet in a clinical scenario.

## Data Availability

Not applicable.

## Abbreviations

CPAP: Continuous positive airway pressure
H-CPAP: Helmet continuous positive airway pressure
NPPV: Noninvasive positive pressure ventilation
CPE: Cardiogenic pulmonary oedema
COVID-19: Coronavirus disease 2019
PEEP: Positive end-expiratory pressure
NIV: Noninvasive ventilatory support
COPD: Chronic obstructive pulmonary disease
