# Supplementary material for: The use of head helmets to deliver noninvasive ventilatory support: a comprehensive review of technical aspects and clinical findings
Source: Crit Care. 2021 Sep 8;25:327. doi: 10.1186/s13054-021-03746-8 (PMC8424168; doi:10.1186/s13054-021-03746-8)
Supplement: Supplementary file 1 — Additional file 1. List of papers retrived by literatire search but excluded from the review. [file 13054_2021_3746_MOESM1_ESM.docx]

**Head Helmets to deliver non-invasive ventilation: a review of technical and clinical aspects**

Andrea Coppadoro^1^ M.D., Elisabetta Zago^1,2^ M.D., Fabio Pavan^1,2^ M.D., Giuseppe Foti^1,2^ M.D., Giacomo Bellani^1,2^ M.D. Ph.D.

^1^ASST Monza, San Gerardo Hospital, Monza, Italy

^2^ Department of Medicine and Surgery, University of Milan-Bicocca, Monza, Italy

Additional File 1

eTABLE 1-Excluded papers

| **Reason for exclusion** | | **Title** | | | | **Authors** | **Citation** |
| --- | --- | --- | --- | --- | --- | --- | --- |
| NOT RELEVANT | | Optimal protection in direct closed head impact | | | | Liu YK, Nikravesh EP, Beck CH. | IEEE Trans Biomed Eng. 1976 Jan;23(1):29-35. doi: 10.1109/tbme.1976.324612. |
| NOT RELEVANT | | An audiometric survey of Navy divers | | | | Brady JI Jr, Summitt JK, Berghage TE. | Undersea Biomed Res. 1976 Mar;3(1):41-7. |
| NOT RELEVANT | | Changes in human intracerebral temperature in response to different methods of brain cooling | | | | Mellergård P. | Neurosurgery. 1992 Oct;31(4):671-7; discussion 677. doi: 10.1227/00006123-199210000-00009. |
| NOT RELEVANT | | Maximum intra-thoracic pressure with anti-G straining maneuvers and positive pressure breathing during +Gz | | | | Buick F, Hartley J, Pecaric M. | Aviat Space Environ Med. 1992 Aug;63(8):670-7. |
| NOT RELEVANT | | The impact of motorcycle helmet use | | | | Offner PJ, Rivara FP, Maier RV. | J Trauma. 1992 May;32(5):636-41; discussion 641-2. doi: 10.1097/00005373-199205000-00016. |
| NOT RELEVANT | | Mandatory bicycle helmet use: experience in Victoria, Australia | | | | Vulcan AP, Cameron MH, Watson WL. | World J Surg. 1992 May-Jun;16(3):389-97. doi: 10.1007/BF02104437. |
| NOT RELEVANT | | Helmet use improves outcomes after motorcycle accidents | | | | Murdock MA, Waxman K. | West J Med. 1991 Oct;155(4):370-2. |
| NOT RELEVANT | | Exposure to fumes in typical New Zealand welding operations | | | | Dryson EW, Rogers DA. | N Z Med J. 1991 Aug 28;104(918):365-7. |
| NOT RELEVANT | | Respiratory effects of benzodiazepine-related drugs in awake rhesus monkeys | | | | Wettstein JG, Teeple ES, Morse WH. | J Pharmacol Exp Ther. 1990 Dec;255(3):1328-34. |
| NOT RELEVANT | | The effectiveness of ice- and Freon-based personal cooling systems during work in fully encapsulating suits in the heat | | | | White MK, Glenn SP, Hudnall J, Rice C, Clark S. | Am Ind Hyg Assoc J. 1991 Mar;52(3):127-35. doi: 10.1080/15298669191364460. |
| NOT RELEVANT | | Attitudes toward bicycle helmet ownership and use by school-age children | | | | DiGuiseppi CG, Rivara FP, Koepsell TD. | Am J Dis Child. 1990 Jan;144(1):83-6. doi: 10.1001/archpedi.1990.02150250093041. |
| NOT RELEVANT | | Respiratory effects of xanthines and adenosine analogs in rhesus monkeys | | | | Howell LL, Morse WH, Spealman RD. | J Pharmacol Exp Ther. 1990 Sep;254(3):786-91. |
| NOT RELEVANT | | [Drug prevention and physical protection in occupational asthma] | | | | Bessot JC. | Allerg Immunol (Paris). 1988 Oct;20(8):293-7. |
| NOT RELEVANT | | Safety precautions for bronchoscopic Nd-YAG laser surgery | | | | Shapshay SM, Beamis JF Jr. | Otolaryngol Head Neck Surg. 1986 Feb;94(2):175-80. doi: 10.1177/019459988609400208. |
| NOT RELEVANT | | Medical and economic parameters of motorcycle-induced trauma | | | | Bried JM, Cordasco FA, Volz RG. | Clin Orthop Relat Res. 1987 Oct;(223):252-6. |
| NOT RELEVANT | | The effect of head and neck suction on G tolerance | | | | Glaister DH, Lenox JB. | Aviat Space Environ Med. 1987 Nov;58(11):1075-81. |
| NOT RELEVANT | | Fire fighter helmet ventilation analysis | | | | Reischl U. | Am Ind Hyg Assoc J. 1986 Sep;47(9):546-51. doi: 10.1080/15298668691390205. |
| NOT RELEVANT | | Evidence that the arterial baroreceptors influence muscle blood flow and not subcutaneous flow in man | | | | Skagen K, Bonde-Petersen F, Henriksen O. | Acta Physiol Scand. 1985 Apr;123(4):405-9. doi: 10.1111/j.1748-1716.1985.tb07607.x. |
| NOT RELEVANT | | Cardiovascular responses to isometric neck muscle contractions: results after dynamic exercise with various headgear loading configurations | | | | Phillips CA, Petrofsky JS. | Aviat Space Environ Med. 1984 Aug;55(8):740-5. |
| NOT RELEVANT | | Workplace protection factor measurements on powered air-purifying respirators at a secondary lead smelter--test protocol | | | | Myers WR, Peach MJ 3rd, Allender J. | Am Ind Hyg Assoc J. 1984 Apr;45(4):236-41. doi: 10.1080/15298668491399712. |
| NOT RELEVANT | | [Significance of the helmet respirator in the treatment of farmer's occupational asthma] | | | | Taivainen A, Tukiainen H, Terho EO, Husman K. | Duodecim. 1994;110(4):423-8. |
| NOT RELEVANT | | Attenuation of hypoxia-induced increases in ventilation by adenosine antagonists in rhesus monkeys | | | | Howell LL, Landrum AM. | Life Sci. 1995;57(8):773-83. doi: 10.1016/0024-3205(95)02005-4. |
| NOT RELEVANT | | Ventilation tubes, swimming and otorrhoea: a New Zealand perspective | | | | Davison MJ, Fields MJ. | N Z Med J. 1993 May 26;106(956):201-3. |
| NOT RELEVANT | | The use of silly putty as an ear plug | | | | Muntz HR. | Arch Otolaryngol Head Neck Surg. 1995 Mar;121(3):354. doi: 10.1001/archotol.1995.01890030080018. |
| NOT RELEVANT | | Application of a valveless anesthesia circuit for deep diving | | | | Zwingelberg KM, Jaeger MJ. | Undersea Hyperb Med. 1994 Dec;21(4):443-58. |
| NOT RELEVANT | | [Discrepancies in assessment of radiological exposure level in miners related to the use of various measurement systems] | | | | Domański T, Chruścielewski W. | Med Pr. 1994;45(1):29-36. |
| NOT RELEVANT | | Acoustic myography in the assessment of human masseter muscle | | | | L'Estrange PR, Rowell J, Stokes MJ. | J Oral Rehabil. 1993 Jul;20(4):353-62. doi: 10.1111/j.1365-2842.1993.tb01618.x. |
| NOT RELEVANT | | Cervical collars: a potential risk to the head-injured patient | | | | Ferguson J, Mardel SN, Beattie TF, Wytch R. | Injury. 1993 Aug;24(7):454-6. doi: 10.1016/0020-1383(93)90148-y. |
| NOT RELEVANT | | Fatal cycling injuries | | | | Noakes TD. | Sports Med. 1995 Nov;20(5):348-62. doi: 10.2165/00007256-199520050-00006. |
| NOT RELEVANT | | Water precautions in children with tympanostomy tubes | | | | Salata JA, Derkay CS. | Arch Otolaryngol Head Neck Surg. 1996 Mar;122(3):276-80. doi: 10.1001/archotol.1996.01890150054010. |
| NOT RELEVANT | | Component analysis and stimulus control assessment of a behavior deceleration treatment package | | | | Cameron MJ, Luiselli JK, Littleton RF Jr, Ferrelli L. | Res Dev Disabil. 1996 May-Jun;17(3):203-15. doi: 10.1016/0891-4222(96)00004-2. |
| NOT RELEVANT | | Nitrous oxide control in the dental operatory: auxiliary exhaust and mask leakage, design, and scavenging flow rate as factors | | | | Crouch KG, Johnston OE. | Am Ind Hyg Assoc J. 1996 Mar;57(3):272-8. doi: 10.1080/15428119691015007. |
| NOT RELEVANT | | The effect of a portable HEPA-filtered body exhaust system on airborne microbial contamination in a conventional operating room | | | | Bohn WW, McKinsey DS, Dykstra M, Koppe S. | Infect Control Hosp Epidemiol. 1996 Jul;17(7):419-22. doi: 10.1086/647332. |
| NOT RELEVANT | | Clothing in laminar-flow operating theatres | | | | Hubble MJ, Weale AE, Perez JV, Bowker KE, MacGowan AP, Bannister GC. | J Hosp Infect. 1996 Jan;32(1):1-7. doi: 10.1016/s0195-6701(96)90159-0. |
| NOT RELEVANT | | Finite-element models of the human head | | | | Voo K, Kumaresan S, Pintar FA, Yoganandan N, Sances A Jr. | Med Biol Eng Comput. 1996 Sep;34(5):375-81. doi: 10.1007/BF02520009. |
| NOT RELEVANT | | Teenagers' attitudes towards bicycle helmets three years after the introduction of mandatory wearing | | | | Finch CF. | Inj Prev. 1996 Jun;2(2):126-30. doi: 10.1136/ip.2.2.126. |
| NOT RELEVANT | | Evaluation of a bicycle helmet giveaway program--Texas, 1995 | | | | Logan P, Leadbetter S, Gibson RE, Schieber R, Branche C, Bender P, Zane D, Humphreys J, Anderson S. | Pediatrics. 1998 Apr;101(4 Pt 1):578-82. doi: 10.1542/peds.101.4.578. |
| NOT RELEVANT | | Some design recommendations to improve comfort in helmets: a case study from China | | | | Abeysekera J, Holmer I, Liu X, Gao C, Wu Z. | J Hum Ergol (Tokyo). 1996 Dec;25(2):145-54. |
| NOT RELEVANT | | Surgical helmet systems and protection of staff and patients | | | |  | Healthc Hazard Mater Manage. 1996 May;9(8):1-4. |
| NOT RELEVANT | | Collaborative assessment: exploring parental injury prevention strategies through bicycle helmet use | | | | Hendrickson SL, Becker H, Compton L. | J Public Health Manag Pract. 1997 Nov;3(6):60-70. doi: 10.1097/00124784-199711000-00013. |
| NOT RELEVANT | | Developing quality measures for adolescent care: validity of adolescents' self-reported receipt of preventive services | | | | Klein JD, Graff CA, Santelli JS, Hedberg VA, Allan MJ, Elster AB. | Health Serv Res. 1999 Apr;34(1 Pt 2):391-404. |
| NOT RELEVANT | | Carbon dioxide accumulation, walking performance, and metabolic cost in the NASA launch and entry suit | | | | Bishop PA, Lee SM, Conza NE, Clapp LL, Moore AD Jr, Williams WJ, Guilliams ME, Greenisen MC. | Aviat Space Environ Med. 1999 Jul;70(7):656-65. |
| NOT RELEVANT | | Preventive services in a health maintenance organization: how well do pediatricians screen and educate adolescent patients? | | | | Halpern-Felsher BL, Ozer EM, Millstein SG, Wibbelsman CJ, Fuster CD, Elster AB, Irwin CE Jr. | Arch Pediatr Adolesc Med. 2000 Feb;154(2):173-9. doi: 10.1001/archpedi.154.2.173. |
| NOT RELEVANT | | Study for verification testing of the helmet-mounted display in the Japanese Experimental Module | | | | Nakajima I, Yamamoto I, Kato H, Inokuchi S, Nemoto M. | J Med Syst. 2000 Feb;24(1):1-9. doi: 10.1023/a:1005456326419. |
| NOT RELEVANT | | Community-based injury prevention interventions | | | | Klassen TP, MacKay JM, Moher D, Walker A, Jones AL. | Future Child. 2000 Spring-Summer;10(1):83-110. |
| NOT RELEVANT | | Sound-level measurements and calculations of safe noise dosage during EPI at 3 T | | | | Foster JR, Hall DA, Summerfield AQ, Palmer AR, Bowtell RW. | J Magn Reson Imaging. 2000 Jul;12(1):157-63. doi: 10.1002/1522-2586(200007)12:1<157::aid-jmri17>3.0.co;2-m. |
| NOT RELEVANT | | A system for quantifying the cooling effectiveness of bicycle helmets | | | | Reid J, Wang EL. | J Biomech Eng. 2000 Aug;122(4):457-60. doi: 10.1115/1.1287163. |
| NOT RELEVANT | | Consequences of nonpenetrating projectile impact on a protected head: study of rear effects of protections | | | | Sarron JC, Caillou JP, Da Cunha J, Allain JC, Trameçon A. | J Trauma. 2000 Nov;49(5):923-9. doi: 10.1097/00005373-200011000-00021. |
| NOT RELEVANT | | Swimming with tympanostomy tubes | | | | Giannoni C. | Arch Otolaryngol Head Neck Surg. 2000 Dec;126(12):1507-8;discussion 1509. |
| NOT RELEVANT | | Isolating the auditory system from acoustic noise during functional magnetic resonance imaging: examination of noise conduction through the ear canal, head, and body | | | | Ravicz ME, Melcher JR. | J Acoust Soc Am. 2001 Jan;109(1):216-31. doi: 10.1121/1.1326083. |
| NOT RELEVANT | | Simulated shuttle egress: comparison of two Space Shuttle protective garments | | | | Lee SM, Bishop PA, Schneider SM, Greenisen MC. | Aviat Space Environ Med. 2001 Feb;72(2):110-4. |
| NOT RELEVANT | | Biomechanics of neurotrauma | | | | Zhang L, Yang KH, King AI. | Neurol Res. 2001 Mar-Apr;23(2-3):144-56. doi: 10.1179/016164101101198488. |
| NOT RELEVANT | | Simulated shuttle egress: role of helmet visor position during approach and landing | | | | Lee SM, Bishop PA, Schneider SM, Clapp LL, Williams WJ, Conza N, Greenisen MC. | Aviat Space Environ Med. 2001 May;72(5):484-9. |
| CASE REPORT/SERIES | | Protective headgear for midwestern agriculture: a limited wear study | | | | Stone JF, Hanna M, Guo C, Imerman P. | J Environ Health. 2001 Mar;63(7):13-9, 21. |
| REVIEW/EDITORIAL | | The respiratory system during resuscitation: a review of the history, risk of infection during assisted ventilation, respiratory mechanics, and ventilation strategies for patients with an unprotected airway | | | | Wenzel V, Idris AH, Dörges V, Nolan JP, Parr MJ, Gabrielli A, Stallinger A, Lindner KH, Baskett PJ. | Resuscitation. 2001 May;49(2):123-34. doi: 10.1016/s0300-9572(00)00349-x. |
| NOT RELEVANT | | Barriers to bicycle helmet use | | | | Finnoff JT, Laskowski ER, Altman KL, Diehl NN. | Pediatrics. 2001 Jul;108(1):E4. doi: 10.1542/peds.108.1.e4. |
| NOT RELEVANT | | Removal of CO2, moisture and heat from ventilated suit under different pressures | | | | Xu G. | Space Med Med Eng (Beijing). 1996 Aug;9(4):251-5. |
| NOT RELEVANT | | Advanced Crew Escape Suit | | | |  | Aerosp Eng. 1995 Sep;15(9):11-4. |
| NOT RELEVANT | | [Human physiological regulation in the closed suit with no ventilation] | | | | Zhang H, Peng YK, Xu GL, Yang F. | Space Med Med Eng (Beijing). 2000 Oct;13(5):332-5. |
| NOT RELEVANT | | Increasing the use of bicycle helmets: lessons from behavioral science | | | | Thompson NJ, Sleet D, Sacks JJ. | Patient Educ Couns. 2002 Mar;46(3):191-7. doi: 10.1016/s0738-3991(01)00212-9. |
| LANGUAGE | | [CPAP in acute pulmonary edema. Our experience of the nurse role in the out of hospital life support] | | | | Sironi S, Brambilla G, Gaiotto M, Donolato F, Foti G, Rossi GP, Pesenti A. | Minerva Anestesiol. 2002 May;68(5):470-4. |
| REVIEW/EDITORIAL | | Use and nursing of the helmet in delivering non invasive ventilation | | | | Scandroglio M, Piccolo U, Mazzone E, Agrati P, Aspesi M, Gamberoni C, Severgnini P, Di Stella R, Chiumello D, Minoja G, Pelosi P. | Minerva Anestesiol. 2002 May;68(5):475-80. |
| LANGUAGE | | [Helmet delivered CPAP for in-patients] | | | | Ferrario D, Lucchini A. | Minerva Anestesiol. 2002 May;68(5):481-4. |
| NOT RELEVANT | | A novel method for chronic measurement of respiratory function in the conscious monkey | | | | Murphy DJ, Renninger JP, Coatney RW. | J Pharmacol Toxicol Methods. 2001 Jul-Aug;46(1):13-20. doi: 10.1016/s1056-8719(01)00159-9. |
| NOT RELEVANT | | Helmet design to facilitate thermoneutrality during forest harvesting | | | | Holland EJ, Laing RM, Lemmon TL, Niven BE. | Ergonomics. 2002 Aug 15;45(10):699-716. doi: 10.1080/00140130210159959. |
| CASE REPORT/SERIES | | Fiberoptic bronchoscopy during noninvasive positive pressure ventilation delivered by helmet | | | | Antonelli M, Pennisi MA, Conti G, Bello G, Maggiore SM, Michetti V, Cavaliere F, Proietti R. | Intensive Care Med. 2003 Jan;29(1):126-9. doi: 10.1007/s00134-002-1554-5. Epub 2002 Nov 29. |
| NOT RELEVANT | | [Noninvasive ventilation in the intensive care unit -- is it still negligible?] | | | | Welte T. | Wien Klin Wochenschr. 2003 Feb 28;115(3-4):89-98. doi: 10.1007/BF03040286. |
| NOT RELEVANT | | "Born to be wild". The effect of the repeal of Florida's mandatory motorcycle helmet-use law on serious injury and fatality rates | | | | Stolzenberg L, D'Alessio SJ. | Eval Rev. 2003 Apr;27(2):131-50. doi: 10.1177/0193841X02250524. |
| REVIEW/EDITORIAL | | Non-invasive ventilation delivered by conventional interfaces and helmet in the emergency department | | | | Pelosi P, Severgnini P, Aspesi M, Gamberoni C, Chiumello D, Fachinetti C, Introzzi L, Antonelli M, Chiaranda M. | Eur J Emerg Med. 2003 Jun;10(2):79-86. doi: 10.1097/00063110-200306000-00002. |
| NOT RELEVANT | | Efficacy and tolerability of non-invasive ventilation delivered via a newly developed helmet in immunosuppressed patients with acute respiratory failure | | | | Rabitsch W, Schellongowski P, Köstler WJ, Stoiser B, Knöbl P, Locker GJ, Sperr W, Burgmann H, Herkner H, Keil F, Frass M, Staudinger T. | Wien Klin Wochenschr. 2003 Sep 15;115(15-16):590-4. doi: 10.1007/BF03040454. |
| NOT RELEVANT | | Evaluation of management of road trauma survivors with brain injury and neurologic disability in Victoria | | | | McDermott FT, Rosenfeld JV, Laidlaw JD, Cordner SM, Tremayne AB; Consultative Committee on Road Traffic Fatalities in Victoria. | J Trauma. 2004 Jan;56(1):137-49. doi: 10.1097/01.TA.0000056163.58047.74. |
| NOT RELEVANT | | Pressure on the face while in the prone position: ProneView versus Prone Positioner | | | | Atwater BI, Wahrenbrock E, Benumof JL, Mazzei WJ. | J Clin Anesth. 2004 Mar;16(2):111-6. doi: 10.1016/j.jclinane.2003.06.001. |
| NOT RELEVANT | | Bicycle helmet ventilation and comfort angle dependence | | | | Brühwiler PA, Ducas C, Huber R, Bishop PA. | Eur J Appl Physiol. 2004 Sep;92(6):698-701. doi: 10.1007/s00421-004-1114-5. |
| NOT RELEVANT | | [Unexplained circulatory collapse after a motorcycle accident] | | | | Kalstad J, Sandvik J, Myrmel T, Bjørsvik G, Nielsen EW. | Tidsskr Nor Laegeforen. 2004 Jun 3;124(11):1523-4. |
| NOT RELEVANT | | Management of ventral hernia after giant exomphalos with external pressure compression using helmet device | | | | Mali VP, Prabhakaran K, Patankar JZ. | J Pediatr Surg. 2004 Aug;39(8):e1-4. doi: 10.1016/j.jpedsurg.2004.04.041. |
| CASE REPORT/SERIES | | Helmet noninvasive ventilation for weaning from mechanical ventilation | | | | Klein M, Weksler N, Bartal C, Gurman GM. | Respir Care. 2004 Sep;49(9):1035-7. |
| NOT RELEVANT | | Dynamic effects of a 9 mm missile on cadaveric skull protected by aramid, polyethylene or aluminum plate: an experimental study | | | | Sarron JC, Dannawi M, Faure A, Caillou JP, Da Cunha J, Robert R. | J Trauma. 2004 Aug;57(2):236-42; discussion 243. doi: 10.1097/01.ta.0000133575.48065.3f. |
| NOT RELEVANT | | The effect of safety hat on thermal responses and working efficiency under a high temperature environment | | | | Kim HE, Park SJ. | J Physiol Anthropol Appl Human Sci. 2004 Sep;23(5):149-53. doi: 10.2114/jpa.23.149. |
| REVIEW/EDITORIAL | | Noninvasive interfaces: should we go to helmets? | | | | Hill NS. | Crit Care Med. 2004 Oct;32(10):2162-3. doi: 10.1097/01.ccm.0000142945.20310.e4. |
| NOT RELEVANT | | An improved motorcycle helmet design for tropical climates | | | | Patel R, Mohan D. | Appl Ergon. 1993 Dec;24(6):427-31. doi: 10.1016/0003-6870(93)90175-9. |
| NOT RELEVANT | | Evaluation of exhalation valves | | | | Kuo YM, Lai CY, Chen CC, Lu BH, Huang SH, Chen CW. | Ann Occup Hyg. 2005 Oct;49(7):563-8. doi: 10.1093/annhyg/mei003. Epub 2005 Feb 16. |
| LANGUAGE | | [A helmet with many advantages] | | | | Burkhalter H. | Krankenpfl Soins Infirm. 2005;98(2):10-3. |
| CASE REPORT/SERIES | | Noninvasive ventilation options in pediatric myasthenia gravis | | | | Piastra M, Conti G, Caresta E, Tempera A, Chiaretti A, Polidori G, Antonelli M. | Paediatr Anaesth. 2005 Aug;15(8):699-702. doi: 10.1111/j.1460-9592.2005.01617.x. |
| NOT RELEVANT | | Efficacy of three face masks in preventing inhalation of airborne contaminants in dental practice | | | | Checchi L, Montevecchi M, Moreschi A, Graziosi F, Taddei P, Violante FS. | J Am Dent Assoc. 2005 Jul;136(7):877-82. doi: 10.14219/jada.archive.2005.0288. |
| NOT RELEVANT | | Manganese exposures during shielded metal arc welding (SMAW) in an enclosed space | | | | Harris MK, Ewing WM, Longo W, DePasquale C, Mount MD, Hatfield R, Stapleton R. | J Occup Environ Hyg. 2005 Aug;2(8):375-82. doi: 10.1080/15459620591007736. |
| NOT RELEVANT | | Respiratory failure after stem cell transplantation: improved outcome with non-invasive ventilation | | | | Rabitsch W, Staudinger T, Locker GJ, Köstler WJ, Laczika K, Frass M, Knoebl P, Greinix HT, Kalhs P, Keil F. | Leuk Lymphoma. 2005 Aug;46(8):1151-7. doi: 10.1080/10428190500097649. |
| NOT RELEVANT | | Biomechanics of the head for Olympic boxer punches to the face | | | | Walilko TJ, Viano DC, Bir CA. | Br J Sports Med. 2005 Oct;39(10):710-9. doi: 10.1136/bjsm.2004.014126. |
| LANGUAGE | | [Description of a new procedure for fiberoptic bronchoscopy during noninvasive ventilation through a nasal mask in patients with acute respiratory failure] | | | | Chiner E, Llombart M, Signes-Costa J, Andreu AL, Gómez-Merino E, Pastor E, Arriero JM. | Arch Bronconeumol. 2005 Dec;41(12):698-701. doi: 10.1016/s1579-2129(06)60337-6. |
| CASE REPORT/SERIES | | Noninvasive ventilation in childhood acute neuromuscular respiratory failure: a pilot study | | | | Piastra M, Antonelli M, Caresta E, Chiaretti A, Polidori G, Conti G. | Respiration. 2006;73(6):791-8. doi: 10.1159/000090777. Epub 2006 Jan 16. |
| LANGUAGE | | [Non-Invasive Mechanical Ventilation in COPD exacerbations] | | | | Macri A, Stoica RT. | Pneumologia. 2005 Jul-Sep;54(3):132-8. |
| LETTER | | Is the helmet different than the face mask in delivering noninvasive ventilation? | | | | Chiumello D. | Chest. 2006 Jun;129(6):1402-3. doi: 10.1378/chest.129.6.1402. |
| REVIEW/EDITORIAL | | Noninvasive ventilation in neuromuscular disease: equipment and application | | | | Hess DR. | Respir Care. 2006 Aug;51(8):896-911; discussion 911-2. |
| NOT RELEVANT | | Heat transfer variations of bicycle helmets | | | | Brühwiler PA, Buyan M, Huber R, Bogerd CP, Sznitman J, Graf SF, Rösgen T. | J Sports Sci. 2006 Sep;24(9):999-1011. doi: 10.1080/02640410500457877. |
| NOT RELEVANT | | All-terrain vehicle-related maxillofacial trauma in the pediatric population | | | | Prigozen JM, Horswell BB, Flaherty SK, Henderson JM, Graham DA, Armistead LM, Habib JH, Lukowski DE. | J Oral Maxillofac Surg. 2006 Sep;64(9):1333-7. doi: 10.1016/j.joms.2006.05.014. |
| NOT RELEVANT | | Effects of information and 50 Hz magnetic fields on cognitive performance and reported symptoms | | | | Nevelsteen S, Legros JJ, Crasson M. | Bioelectromagnetics. 2007 Jan;28(1):53-63. doi: 10.1002/bem.20265. |
| NOT RELEVANT | | Water precautions and ear surgery: evidence and practice in the UK | | | | Basu S, Georgalas C, Sen P, Bhattacharyya AK. | J Laryngol Otol. 2007 Jan;121(1):9-14. doi: 10.1017/S0022215106003136. Epub 2006 Nov 14. |
| NOT RELEVANT | | Effect of footwear and orthotic devices on stress reduction and soft tissue strain of the neuropathic foot | | | | Lott DJ, Hastings MK, Commean PK, Smith KE, Mueller MJ. | Clin Biomech (Bristol, Avon). 2007 Mar;22(3):352-9. doi: 10.1016/j.clinbiomech.2006.10.010. Epub 2006 Dec 19. |
| REVIEW/EDITORIAL | | Lung mechanics at the bedside: make it simple | | | | Lucangelo U, Bernabè F, Blanch L. | Curr Opin Crit Care. 2007 Feb;13(1):64-72. doi: 10.1097/MCC.0b013e32801162df. |
| LANGUAGE | | [Should a helmet be used to deliver noninvasive ventilation?] | | | | Vignaux L, Tassaux D, Jolliet P. | Rev Med Suisse. 2006 Dec 13;2(91):2860-5. |
| LANGUAGE | | [Initial pediatric trauma care and cardiopulmonary resuscitation] | | | | Domínguez Sampedro P, Cañadas Palazón S, de Lucas García N, Balcells Ramírez J, Martínez Ibáñez V. | An Pediatr (Barc). 2006 Dec;65(6):586-606. doi: 10.1016/s1695-4033(06)70255-7. |
| REVIEW/EDITORIAL | | Noninvasive ventilation | | | | Barreiro TJ, Gemmel DJ. | Crit Care Clin. 2007 Apr;23(2):201-22, ix. doi: 10.1016/j.ccc.2006.11.015. |
| NOT RELEVANT | | The mask for noninvasive ventilation: principles of design and effects on aerosol delivery | | | | Hess DR. | J Aerosol Med. 2007;20 Suppl 1:S85-98; discussion S98-9. doi: 10.1089/jam.2007.0574. |
| NOT RELEVANT | | Longitudinal study on potential neurotoxic effects of aluminium: I. Assessment of exposure and neurobehavioural performance of Al welders in the train and truck construction industry over 4 years | | | | Kiesswetter E, Schäper M, Buchta M, Schaller KH, Rossbach B, Scherhag H, Zschiesche W, Letzel S. | Int Arch Occup Environ Health. 2007 Oct;81(1):41-67. doi: 10.1007/s00420-007-0191-2. Epub 2007 May 24. |
| LETTER | | Intrapulmonary percussive ventilation improves the outcomes of helmet ventilation | | | | Ntoumenopoulos G. | Aust J Physiother. 2007;53(2):129. doi: 10.1016/s0004-9514(07)70046-6. |
| NOT RELEVANT | | Particulate and gaseous emissions when welding aluminum alloys | | | | Cole H, Epstein S, Peace J. | J Occup Environ Hyg. 2007 Sep;4(9):678-87. doi: 10.1080/15459620701516162. |
| NOT RELEVANT | | Quantification of ventilation characteristics of a helmet | | | | Van Brecht A, Nuyttens D, Aerts JM, Quanten S, De Bruyne G, Berckmans D. | Appl Ergon. 2008 May;39(3):332-41. doi: 10.1016/j.apergo.2007.08.003. Epub 2007 Oct 24. |
| LETTER | | Noninvasive positive-pressure ventilation in postoperative hypoxemic respiratory failure--with a helmet? | | | | Kacmarek RM. | Respir Care. 2007 Nov;52(11):1451-3. |
| NOT RELEVANT | | Noninvasive positive-pressure ventilation with different interfaces in patients with respiratory failure after abdominal surgery: a matched-control study | | | | Conti G, Cavaliere F, Costa R, Craba A, Catarci S, Festa V, Proietti R, Antonelli M. | Respir Care. 2007 Nov;52(11):1463-71. |
| REVIEW/EDITORIAL | | Noninvasive positive-pressure ventilation in acute respiratory failure | | | | Peñuelas O, Frutos-Vivar F, Esteban A. | CMAJ. 2007 Nov 6;177(10):1211-8. doi: 10.1503/cmaj.060147. |
| CASE REPORT/SERIES | | Occurrence of pneumothorax during noninvasive positive pressure ventilation through a helmet | | | | Carron M, Gagliardi G, Michielan F, Freo U, Ori C. | J Clin Anesth. 2007 Dec;19(8):632-5. doi: 10.1016/j.jclinane.2007.04.010. |
| NOT RELEVANT | | A study of the response of the human cadaver head to impact | | | | Hardy WN, Mason MJ, Foster CD, Shah CS, Kopacz JM, Yang KH, King AI, Bishop J, Bey M, Anderst W, Tashman S. | Stapp Car Crash J. 2007 Oct;51:17-80. |
| NOT RELEVANT | | Pacific Northwest survey: posttympanostomy tube water precautions | | | | Poss JM, Boseley ME, Crawford JV. | Arch Otolaryngol Head Neck Surg. 2008 Feb;134(2):133-5. doi: 10.1001/archoto.2007.25. |
| NOT RELEVANT | | Efficacy of body ventilation system for reducing strain in warm and hot climates | | | | Chinevere TD, Cadarette BS, Goodman DA, Ely BR, Cheuvront SN, Sawka MN. | Eur J Appl Physiol. 2008 Jun;103(3):307-14. doi: 10.1007/s00421-008-0707-9. Epub 2008 Mar 8. |
| NOT RELEVANT | | Human subject testing of leakage in a loose-fitting PAPR | | | | Johnson AT, Koh FC, Jamshidi S, Rehak TE. | J Occup Environ Hyg. 2008 May;5(5):325-9. doi: 10.1080/15459620801996819. |
| LANGUAGE | | [Noninvasive ventilation through a helmet following facial reconstruction surgery] | | | | Redondo FJ, Madrazo M, Villazala R, Bernal G. | Rev Esp Anestesiol Reanim. 2008 Feb;55(2):128-30. doi: 10.1016/s0034-9356(08)70526-7. |
| CASE REPORT/SERIES | | Treatment of cardiogenic pulmonary oedema by helmet-delivered non-invasive pressure support ventilation in children with scorpion sting envenomation | | | | Yildizdas D, Yilmaz HL, Erdem S. | Ann Acad Med Singap. 2008 Mar;37(3):230-4. |
| REVIEW/EDITORIAL | | The use of helmets to deliver non-invasive continuous positive airway pressure in hypoxemic acute respiratory failure | | | | Bellani G, Patroniti N, Greco M, Foti G, Pesenti A. | Minerva Anestesiol. 2008 Nov;74(11):651-6. Epub 2008 Jul 18. |
| LANGUAGE | | [Application of CPAP improves oxygenation during normobaric and hypobaric hypoxia] | | | | Koch R, Punter E, Gatterer H, Flatz M, Faulhaber M, Burtscher M. | Wien Med Wochenschr. 2008;158(5-6):156-9. doi: 10.1007/s10354-007-0502-y. |
| REVIEW/EDITORIAL | | Noninvasive ventilation in infants and children | | | | Kissoon N, Adderley R. | Minerva Pediatr. 2008 Apr;60(2):211-8. |
| NOT RELEVANT | | Helmet ventilation and carbon dioxide rebreathing: effects of adding a leak at the helmet ports | | | | Racca F, Appendini L, Gregoretti C, Varese I, Berta G, Vittone F, Ferreyra G, Stra E, Ranieri VM. | Intensive Care Med. 2008 Aug;34(8):1461-8. doi: 10.1007/s00134-008-1120-x. Epub 2008 May 6. |
| REVIEW/EDITORIAL | | Neural trigger and cycling off during helmet pressure support ventilation: the epitome of the perfect patient ventilator interaction? | | | | Vargas F. | Intensive Care Med. 2008 Sep;34(9):1562-4. doi: 10.1007/s00134-008-1164-y. Epub 2008 May 30. |
| NOT RELEVANT | | Intralesional steroid injection for the management of otohematoma | | | | Im GJ, Chae SW, Choi J, Kim YS, Kim WJ, Jung HH. | Otolaryngol Head Neck Surg. 2008 Jul;139(1):115-9. doi: 10.1016/j.otohns.2008.01.006. |
| LETTER | | Noise levels during neonatal helmet CPAP | | | | Trevisanuto D, Camiletti L, Udilano A, Doglioni N, Zanardo V. | Arch Dis Child Fetal Neonatal Ed. 2008 Sep;93(5):F396-7. doi: 10.1136/adc.2008.140715. |
| LANGUAGE | | [Interfaces for pediatric noninvasive ventilation (excluding neonate)] | | | | Noizet-Yverneau O, Leclerc F, Santerne B, Akhavi A, Pomédio M, Saad S, Dessioux E, Morville P. | Arch Pediatr. 2008 Oct;15(10):1549-59. doi: 10.1016/j.arcped.2008.07.017. Epub 2008 Sep 30. |
| NOT RELEVANT | | The "six sigma approach" to the operating room environment and infection | | | | Thiele RH, Huffmyer JL, Nemergut EC. | Best Pract Res Clin Anaesthesiol. 2008 Sep;22(3):537-52. doi: 10.1016/j.bpa.2008.06.002. |
| NOT RELEVANT | | The skeletal kinematics of lung ventilation in three basal bird taxa (emu, tinamou, and guinea fowl) | | | | Claessens LP. | J Exp Zool A Ecol Genet Physiol. 2009 Oct 1;311(8):586-99. doi: 10.1002/jez.501. |
| REVIEW/EDITORIAL | | Interfaces and humidification for noninvasive mechanical ventilation | | | | Nava S, Navalesi P, Gregoretti C. | Respir Care. 2009 Jan;54(1):71-84. |
| NOT RELEVANT | | Factors affecting anatomical region of injury, severity, and mortality for road trauma in a high-income developing country: lessons for prevention | | | | Eid HO, Barss P, Adam SH, Torab FC, Lunsjo K, Grivna M, Abu-Zidan FM. | Injury. 2009 Jul;40(7):703-7. doi: 10.1016/j.injury.2008.07.012. Epub 2008 Dec 30. |
| CASE REPORT/SERIES | | A successful therapy of high-altitude pulmonary edema with a CPAP helmet on Lenin Peak | | | | Koch RO, Hinterhuber L, Faulhaber M, Gatterer H, Graupner S, Muenzel K, Burtscher M. | Clin J Sport Med. 2009 Jan;19(1):72-3. doi: 10.1097/JSM.0b013e3181915cce. |
| NOT RELEVANT | | Understanding reasons for non-compliance in motorcycle helmet use among adolescents in Greece | | | | Germeni E, Lionis C, Davou B, Petridou ET. | Inj Prev. 2009 Feb;15(1):19-23. doi: 10.1136/ip.2008.019356. |
| NOT RELEVANT | | Evaluation of the biofidelity of FMVSS No. 218 injury criteria | | | | Rigby P, Chan P. | Traffic Inj Prev. 2009 Apr;10(2):170-7. doi: 10.1080/15389580802607796. |
| NOT RELEVANT | | Preventive care for adolescents: few get visits and fewer get services | | | | Irwin CE Jr, Adams SH, Park MJ, Newacheck PW. | Pediatrics. 2009 Apr;123(4):e565-72. doi: 10.1542/peds.2008-2601. |
| NOT RELEVANT | | Helmet with specific settings versus facemask for noninvasive ventilation | | | | Vargas F, Thille A, Lyazidi A, Campo FR, Brochard L. | Crit Care Med. 2009 Jun;37(6):1921-8. doi: 10.1097/CCM.0b013e31819fff93. |
| CASE REPORT/SERIES | | Helmet ventilation for acute respiratory failure and nasal skin breakdown in neuromuscular disorders | | | | Racca F, Appendini L, Berta G, Barberis L, Vittone F, Gregoretti C, Ferreyra G, Urbino R, Ranieri VM. | Anesth Analg. 2009 Jul;109(1):164-7. doi: 10.1213/ane.0b013e3181a1f708. Epub 2009 May 13. |
| LETTER | | Helmet to deliver noninvasive ventilation: "Handle with care" | | | | Nava S, Navalesi P. | Crit Care Med. 2009 Jun;37(6):2111-3. doi: 10.1097/CCM.0b013e3181a5e6b5. |
| REVIEW/EDITORIAL | | Noninvasive ventilation in postoperative care of lung transplant recipients | | | | Feltracco P, Serra E, Barbieri S, Milevoj M, Furnari M, Rizzi S, Rea F, Marulli G, Ori C. | Transplant Proc. 2009 May;41(4):1339-44. doi: 10.1016/j.transproceed.2009.02.048. |
| NOT RELEVANT | | Concussion in professional football: animal model of brain injury--part 15 | | | | Viano DC, Hamberger A, Bolouri H, Säljö A. | Neurosurgery. 2009 Jun;64(6):1162-73; discussion 1173. doi: 10.1227/01.NEU.0000345863.99099.C7. |
| NOT RELEVANT | | Auditory evoked magnetic fields in children with functional hearing loss | | | | Yoshizaki N, Kawase T, Nakasato N, Kanno A, Okitsu T, Sunose H, Kobayashi T. | Int J Pediatr Otorhinolaryngol. 2009 Oct;73(10):1368-72. doi: 10.1016/j.ijporl.2009.06.015. Epub 2009 Jul 23. |
| NOT RELEVANT | | Creation of a head and face protection device for children undergoing procedures in prone position | | | | Wenk M, Pöpping D, Henning M, Wenk M, Liljenqvist U, Möllmann M. | Paediatr Anaesth. 2009 Jun;19(6):587-92. doi: 10.1111/j.1460-9592.2009.03008.x. |
| NOT RELEVANT | | 300 all-terrain vehicle crashes: an East Tennessee trauma center's experience | | | | Testerman GM. | Tenn Med. 2009 Aug;102(8):45-7. |
| NOT RELEVANT | | The facial integument of centrosaurine ceratopsids: morphological and histological correlates of novel skin structures | | | | Hieronymus TL, Witmer LM, Tanke DH, Currie PJ. | Anat Rec (Hoboken). 2009 Sep;292(9):1370-96. doi: 10.1002/ar.20985. |
| CASE REPORT/SERIES | | Helmet-delivered continuous positive airway pressure with heliox in respiratory syncytial virus bronchiolitis | | | | Mayordomo-Colunga J, Medina A, Rey C, Concha A, Los Arcos M, Menéndez S. | Acta Paediatr. 2010 Feb;99(2):308-11. doi: 10.1111/j.1651-2227.2009.01529.x. Epub 2009 Oct 7. |
| NOT RELEVANT | | Incidence of otitis media in children with deformational plagiocephaly | | | | Purzycki A, Thompson E, Argenta L, David L. | J Craniofac Surg. 2009 Sep;20(5):1407-11. doi: 10.1097/SCS.0b013e3181aee369. |
| CASE REPORT | | Long-term use of neonatal helmet-CPAP: a case report | | | | Doglioni N, Micaglio M, Zanardo V, Trevisanuto D. | Minerva Anestesiol. 2009 Dec;75(12):750-3. |
| NOT RELEVANT | | Manganese, iron, and total particulate exposures to welders | | | | Flynn MR, Susi P. | J Occup Environ Hyg. 2010 Feb;7(2):115-26. doi: 10.1080/15459620903454600. |
| NOT RELEVANT | | Noninvasive functional MRI in alert monkeys | | | | Srihasam K, Sullivan K, Savage T, Livingstone MS. | Neuroimage. 2010 May 15;51(1):267-73. doi: 10.1016/j.neuroimage.2010.01.082. Epub 2010 Jan 29. |
| NOT RELEVANT | | The American football uniform: uncompensable heat stress and hyperthermic exhaustion | | | | Armstrong LE, Johnson EC, Casa DJ, Ganio MS, McDermott BP, Yamamoto LM, Lopez RM, Emmanuel H. | J Athl Train. 2010 Mar-Apr;45(2):117-27. doi: 10.4085/1062-6050-45.2.117. |
| NOT RELEVANT | | PLOD1-Related Kyphoscoliotic Ehlers-Danlos Syndrome | | | | Yeowell HN, Steinmann B. | 2000 Feb 2 [updated 2018 Oct 18]. In: Adam MP, Ardinger HH, Pagon RA, Wallace SE, Bean LJH, Mirzaa G, Amemiya A, editors. GeneReviews(®) [Internet]. Seattle (WA): University of Washington, Seattle; 1993–2021. |
| REVIEW/EDITORIAL | | What are the current indications for noninvasive ventilation in children? | | | | Calderini E, Chidini G, Pelosi P. | Curr Opin Anaesthesiol. 2010 Jun;23(3):368-74. doi: 10.1097/ACO.0b013e328339507b. |
| NOT RELEVANT | | Spinal column and spinal cord injuries in mountain bikers: a 13-year review | | | | Dodwell ER, Kwon BK, Hughes B, Koo D, Townson A, Aludino A, Simons RK, Fisher CG, Dvorak MF, Noonan VK. | Am J Sports Med. 2010 Aug;38(8):1647-52. doi: 10.1177/0363546510365532. Epub 2010 May 20. |
| LANGUAGE | | [Place of non-invasive ventilation in patients with COPD exacerbation] | | | | Płusa T. | Pol Merkur Lekarski. 2010 Apr;28(166):315-8. |
| NOT RELEVANT | | Effects of turning on skin-bed interface pressures in healthy adults | | | | Peterson MJ, Schwab W, van Oostrom JH, Gravenstein N, Caruso LJ. | J Adv Nurs. 2010 Jul;66(7):1556-64. doi: 10.1111/j.1365-2648.2010.05292.x. Epub 2010 May 21. |
| NOT RELEVANT | | Physiological effects of boot weight and design on men and women firefighters | | | | Turner NL, Chiou S, Zwiener J, Weaver D, Spahr J. | J Occup Environ Hyg. 2010 Aug;7(8):477-82. doi: 10.1080/15459624.2010.486285. |
| NOT RELEVANT | | Quantifying exposure risk: surgical masks and respirators | | | | Diaz KT, Smaldone GC. | Am J Infect Control. 2010 Sep;38(7):501-8. doi: 10.1016/j.ajic.2010.06.002. |
| REVIEW/EDITORIAL | | Non-invasive ventilation in pediatric intensive care | | | | Gregoretti C, Pelosi P, Chidini G, Bignamini E, Calderini E. | Minerva Pediatr. 2010 Oct;62(5):437-58. |
| NOT RELEVANT | | Review of occupational hazards associated with aquaculture | | | | Myers ML. | J Agromedicine. 2010 Oct;15(4):412-26. doi: 10.1080/1059924X.2010.512854. |
| NOT RELEVANT | | Thermal perception of ventilation changes in full-face motorcycle helmets: subject and manikin study | | | | Bogerd CP, Rossi RM, Brühwiler PA. | Ann Occup Hyg. 2011 Mar;55(2):192-201. doi: 10.1093/annhyg/meq074. Epub 2010 Oct 19. |
| LANGUAGE | | [Usefulness of helmet type mask in the treatment of postoperative atelectasis in a child: a case report] | | | | Takinami Y. | Masui. 2010 Oct;59(10):1271-2. |
| LANGUAGE | | [A case report of successful treatment of a child with respiratory distress using non-invasive continuous positive airway pressure via helmet] | | | | Kono R, Taga N, Okada O, Otsuka Y, Sato Y, Takeuchi M, Seo N. | Masui. 2010 Oct;59(10):1284-6. |
| CASE REPORT | | Helmet-delivered heliox-CPAP in severe upper airway obstruction caused by PHACES syndrome | | | | Vivanco-Allende A, Mayordomo-Colunga J, Coca-Pelaz A, Rey C, Medina A. | Pediatr Pulmonol. 2011 Mar;46(3):306-8. doi: 10.1002/ppul.21367. Epub 2010 Oct 21. |
| NOT RELEVANT | | In silico investigation of intracranial blast mitigation with relevance to military traumatic brain injury | | | | Nyein MK, Jason AM, Yu L, Pita CM, Joannopoulos JD, Moore DF, Radovitzky RA. | Proc Natl Acad Sci U S A. 2010 Nov 30;107(48):20703-8. doi: 10.1073/pnas.1014786107. Epub 2010 Nov 22. |
| NOT RELEVANT | | Individual earmuff during reconstruction of the auricle | | | | Liu X, Pan B, Jiang H, Huang C, Zhuang H. | J Craniofac Surg. 2010 Nov;21(6):1975-6. doi: 10.1097/SCS.0b013e3181f502e6. |
| LANGUAGE | | [Assembly and management of helmet-CPAP in infants and children with acute respiratory insufficiency] | | | | Pérez González S, Mayordomo Colunga J, Rey Galán C, Martín Abad M, Medina Villanueva A, Vázquez Álvarez ML. | Enferm Intensiva. 2011 Apr-Jun;22(2):60-4. doi: 10.1016/j.enfi.2010.08.001. Epub 2011 Jan 21. |
| NOT RELEVANT | | Novice riders and the predictors of riding without motorcycle protective clothing | | | | de Rome L, Ivers R, Haworth N, Heritier S, Du W, Fitzharris M. | Accid Anal Prev. 2011 May;43(3):1095-103. doi: 10.1016/j.aap.2010.12.018. Epub 2011 Jan 17. |
| NOT RELEVANT | | The assessment of airway maneuvers and interventions in university Canadian football, ice hockey, and soccer players | | | | Delaney JS, Al-Kashmiri A, Baylis PJ, Troutman T, Aljufaili M, Correa JA. | J Athl Train. 2011 Mar-Apr;46(2):117-25. doi: 10.4085/1062-6050-46.2.117. |
| LANGUAGE | | [The comfort of patients ventilated with the Helmet Bundle] | | | | Lucchini A, Valsecchi D, Elli S, Doni V, Corsaro P, Tundo P, Re R, Foti G, Manici M. | Assist Inferm Ric. 2010 Oct-Dec;29(4):174-83. |
| CASE REPORT/SERIES | | Successful application of helmet non-invasive ventilation in a parturient with acute respiratory distress syndrome | | | | Frassanito L, Draisci G, Pinto R, Maviglia R, Maggiore SM. | Minerva Anestesiol. 2011 Nov;77(11):1121-3. Epub 2011 Apr 14. |
| NOT RELEVANT | | Distinguishing realistic military blasts from firecrackers in mitigation studies of blast-induced traumatic brain injury | | | | Moss WC, King MJ, Blackman EG. | Proc Natl Acad Sci U S A. 2011 Apr 26;108(17):E82; author reply E83. doi: 10.1073/pnas.1101671108. Epub 2011 Apr 18. |
| LANGUAGE | | [Noninvasive mechanical ventilation in severe pneumonia due to H1N1 virus] | | | | Belenguer-Muncharaz A, Reig-Valero R, Altaba-Tena S, Casero-Roig P, Ferrándiz-Sellés A. | Med Intensiva. 2011 Nov;35(8):470-7. doi: 10.1016/j.medin.2011.04.001. Epub 2011 May 19. |
| LANGUAGE | | [Comparison of non-invasive mechanical ventilation with helmet or face mask in patients with acute exacerbation of chronic obstructive pulmonary disease] | | | | Ali A, Türkmen A, Turgut N, Altan A, Sari T. | Tuberk Toraks. 2011;59(2):146-52. doi: 10.5578/tt.738. |
| NOT RELEVANT | | Sterile surgical helmet system in elective total hip and knee arthroplasty | | | | Singh VK, Hussain S, Javed S, Singh I, Mulla R, Kalairajah Y. | J Orthop Surg (Hong Kong). 2011 Aug;19(2):234-7. doi: 10.1177/230949901101900222. |
| NOT RELEVANT | | Aeroacoustic sources of motorcycle helmet noise | | | | Kennedy J, Adetifa O, Carley M, Holt N, Walker I. | J Acoust Soc Am. 2011 Sep;130(3):1164-72. doi: 10.1121/1.3621097. |
| NOT RELEVANT | | Comfort effects of a new car headrest with neck support | | | | Franz M, Durt A, Zenk R, Desmet PM. | Appl Ergon. 2012 Mar;43(2):336-43. doi: 10.1016/j.apergo.2011.06.009. Epub 2011 Sep 25. |
| NOT RELEVANT | | Development of a multimodal blast sensor for measurement of head impact and over-pressurization exposure | | | | Chu JJ, Beckwith JG, Leonard DS, Paye CM, Greenwald RM. | Ann Biomed Eng. 2012 Jan;40(1):203-12. doi: 10.1007/s10439-011-0410-6. Epub 2011 Oct 13. |
| NOT RELEVANT | | Pharmacokinetics of long-acting ceftiofur crystalline-free acid in helmeted guineafowl (Numida meleagris) after a single intramuscular injection | | | | Wojick KB, Langan JN, Adkesson MJ, Cox SK, Gamble KC. | Am J Vet Res. 2011 Nov;72(11):1514-8. doi: 10.2460/ajvr.72.11.1514. |
| NOT RELEVANT | | Animal model for sport-related concussion; ICP and cognitive function | | | | Bolouri H, Säljö A, Viano DC, Hamberger A. | Acta Neurol Scand. 2012 Apr;125(4):241-7. doi: 10.1111/j.1600-0404.2011.01614.x. Epub 2011 Oct 29. |
| NOT RELEVANT | | Non-legislative interventions for the promotion of cycle helmet wearing by children | | | | Owen R, Kendrick D, Mulvaney C, Coleman T, Royal S. | Cochrane Database Syst Rev. 2011 Nov 9;2011(11):CD003985. doi: 10.1002/14651858.CD003985.pub3. |
| LANGUAGE | | [Noninvasive mechanical ventilation with a helmet in a patient with acute respiratory failure due to alveolar bleeding (Wegener granulomatosis)] | | | | Baladrón V, Redondo FJ, Collar LG, Bernal G. | Rev Esp Anestesiol Reanim. 2011 Oct;58(8):525-6. doi: 10.1016/s0034-9356(11)70130-x. |
| NOT RELEVANT | | Headform and N95 filtering facepiece respirator interaction: contact pressure simulation and validation | | | | Lei Z, Yang JJ, Zhuang Z. | J Occup Environ Hyg. 2012;9(1):46-58. doi: 10.1080/15459624.2011.635130. |
| NOT RELEVANT | | A multidimensional approach to the generation of helmets' design criteria: a preliminar study | | | | Alemany S, Olaso J, Nacher B, Gil M, Hernández A, Pizá M, Solves C. | Work. 2012;41 Suppl 1:4031-7. doi: 10.3233/WOR-2012-0067-4031. |
| NOT RELEVANT | | Investigating helmet promotion for cyclists: results from a randomised study with observation of behaviour, using a semi-automatic video system | | | | Constant A, Messiah A, Felonneau ML, Lagarde E. | PLoS One. 2012;7(2):e31651. doi: 10.1371/journal.pone.0031651. Epub 2012 Feb 15. |
| NOT RELEVANT | | Costs associated with helmet use in motorcycle crashes: the cost of not wearing a helmet | | | | Heldt KA, Renner CH, Boarini DJ, Swegle JR. | Traffic Inj Prev. 2012;13(2):144-9. doi: 10.1080/15389588.2011.637252. |
| NOT RELEVANT | | Impact of two particle measurement techniques on the determination of N95 class respirator filtration performance against ultrafine particles | | | | Mostofi R, Noël A, Haghighat F, Bahloul A, Lara J, Cloutier Y. | J Hazard Mater. 2012 May 30;217-218:51-7. doi: 10.1016/j.jhazmat.2012.02.058. Epub 2012 Mar 2. |
| NOT RELEVANT | | Attenuation of blast pressure behind ballistic protective vests | | | | Wood GW, Panzer MB, Shridharani JK, Matthews KA, Capehart BP, Myers BS, Bass CR. | Inj Prev. 2013 Feb;19(1):19-25. doi: 10.1136/injuryprev-2011-040277. Epub 2012 Apr 29. |
| NOT RELEVANT | | Retrospective review of all-terrain vehicle accidents in Alberta | | | | Pelletier JS, McKee J, Ozegovic D, Widder S. | Can J Surg. 2012 Aug;55(4):249-53. doi: 10.1503/cjs.036210. |
| NOT RELEVANT | | Quantification of head sweating during rest and exercise in the heat | | | | O'Brien C, Cadarette BS. | Eur J Appl Physiol. 2013 Mar;113(3):735-41. doi: 10.1007/s00421-012-2482-x. Epub 2012 Sep 1. |
| NOT RELEVANT | | Flexible electrode belt for EIT using nanofiber web dry electrodes | | | | Oh TI, Kim TE, Yoon S, Kim KJ, Woo EJ, Sadleir RJ. | Physiol Meas. 2012 Oct;33(10):1603-16. doi: 10.1088/0967-3334/33/10/1603. Epub 2012 Sep 4. |
| NOT RELEVANT | | Dispersion and exposure to a cough-generated aerosol in a simulated medical examination room | | | | Lindsley WG, King WP, Thewlis RE, Reynolds JS, Panday K, Cao G, Szalajda JV. | J Occup Environ Hyg. 2012;9(12):681-90. doi: 10.1080/15459624.2012.725986. |
| REVIEW/EDITORIAL | | Recent advances in interfaces for non-invasive ventilation: from bench studies to practical issues | | | | Sferrazza Papa GF, Di Marco F, Akoumianaki E, Brochard L. | Minerva Anestesiol. 2012 Oct;78(10):1146-53. |
| NOT RELEVANT | | Forehead-mounted reflectance oximetry for in-cockpit hypoxia early detection and warning | | | | Simmons RG, Chandler JF, Horning DS. | Aviat Space Environ Med. 2012 Nov;83(11):1067-76. doi: 10.3357/asem.3156.2012. |
| NOT RELEVANT | | A computational study of influence of helmet padding materials on the human brain under ballistic impacts | | | | Salimi Jazi M, Rezaei A, Karami G, Azarmi F, Ziejewski M. | Comput Methods Biomech Biomed Engin. 2014;17(12):1368-82. doi: 10.1080/10255842.2012.748755. Epub 2013 Jan 3. |
| NOT RELEVANT | | Efficacy of commercial earplugs in preventing water intrusion during swimming | | | | Mahboubi H, Lee A, Kiumehr S, Zardouz S, Shahriari S, Djalilian HR. | Otolaryngol Head Neck Surg. 2013 Mar;148(3):415-9. doi: 10.1177/0194599812471798. Epub 2013 Jan 2. |
| NOT RELEVANT | | Etiologies of pediatric craniofacial injuries: a comparison of injuries involving all-terrain vehicles and golf carts | | | | White LC, McKinnon BJ, Hughes CA. | Int J Pediatr Otorhinolaryngol. 2013 Mar;77(3):414-7. doi: 10.1016/j.ijporl.2012.11.043. Epub 2013 Jan 6. |
| LETTER | | Effectiveness of helmet non-invasive ventilation with external PEEP valves: key remains inside the helmet | | | | Esquinas AM, Consentini R, Pravinkumar E, Dikmen Y. | Minerva Anestesiol. 2013 Jun;79(6):697-8. Epub 2013 Feb 28. |
| CASE REPORT/SERIES | | Respiratory failure in a patient with dermatomyositis | | | | Salimbene I, Leli I, Valente S. | Multidiscip Respir Med. 2013 Mar 27;8(1):27. doi: 10.1186/2049-6958-8-27. |
| NOT RELEVANT | | Respiratory source control versus receiver protection: impact of facemask fit | | | | Mansour MM, Smaldone GC. | J Aerosol Med Pulm Drug Deliv. 2013 Jun;26(3):131-7. doi: 10.1089/jamp.2012.0998. Epub 2013 Apr 1. |
| NOT RELEVANT | | Blood-brain barrier dysfunction after primary blast injury in vitro | | | | Hue CD, Cao S, Haider SF, Vo KV, Effgen GB, Vogel E 3rd, Panzer MB, Bass CR, Meaney DF, Morrison B 3rd. | J Neurotrauma. 2013 Oct 1;30(19):1652-63. doi: 10.1089/neu.2012.2773. Epub 2013 Aug 28. |
| NOT RELEVANT | | An integrated helmet and neck support (iHANS) for racing car drivers: a biomechanical feasibility study | | | | Newman JA, Withnall C, Wonnacott M. | Stapp Car Crash J. 2012 Oct;56:469-84. |
| LETTER | | Complications following pulmonary lobectomy: the role of helmet noninvasive ventilation | | | | Unnikrishnan R, John PJ, Shenoy A. | Respir Care. 2013 May;58(5):e64. doi: 10.4187/respcare.02248. |
| REVIEW/EDITORIAL | | Noninvasive mechanical ventilation and helmet after lung resection: oxygenation improvement: a small step or a large step? | | | | Esquinas AM, Papadakos PJ. | Respir Care. 2013 May;58(5):e65-6. doi: 10.4187/respcare.02333. |
| REVIEW/EDITORIAL | | Clinical review: Helmet and non-invasive mechanical ventilation in critically ill patients | | | | Esquinas Rodriguez AM, Papadakos PJ, Carron M, Cosentini R, Chiumello D. | Crit Care. 2013 Apr 25;17(2):223. doi: 10.1186/cc11875. |
| NOT RELEVANT | | Helmets prevent motorcycle injuries with significant economic benefits | | | | Philip AF, Fangman W, Liao J, Lilienthal M, Choi K. | Traffic Inj Prev. 2013;14(5):496-500. doi: 10.1080/15389588.2012.727109. |
| NOT RELEVANT | | Reduction in welding fume and metal exposure of stainless steel welders: an example from the WELDOX study | | | | Lehnert M, Weiss T, Pesch B, Lotz A, Zilch-Schöneweis S, Heinze E, Van Gelder R, Hahn JU, Brüning T; WELDOX Study Group. | Int Arch Occup Environ Health. 2014 Jul;87(5):483-92. doi: 10.1007/s00420-013-0884-7. Epub 2013 May 30. |
| NOT RELEVANT | | Mechanical ventilation in ICUs in Poland: a multi-center point-prevalence study | | | | Kübler A, Maciejewski D, Adamik B, Kaczorowska M. | Med Sci Monit. 2013 Jun 3;19:424-9. doi: 10.12659/MSM.883930. |
| NOT RELEVANT | | Methodology to determine skull bone and brain responses from ballistic helmet-to-head contact loading using experiments and finite element analysis | | | | Pintar FA, Philippens MM, Zhang J, Yoganandan N. | Med Eng Phys. 2013 Nov;35(11):1682-7. doi: 10.1016/j.medengphy.2013.04.015. Epub 2013 Jun 20. |
| NOT RELEVANT | | On the accuracy of the Head Impact Telemetry (HIT) System used in football helmets | | | | Jadischke R, Viano DC, Dau N, King AI, McCarthy J. | J Biomech. 2013 Sep 3;46(13):2310-5. doi: 10.1016/j.jbiomech.2013.05.030. Epub 2013 Jul 26. |
| LETTER | | The authors respond to: Complications following pulmonary lobectomy: the role of helmet noninvasive ventilation | | | | Ampollini L, Barbagallo M, Ziegler S, Ortu A. | Respir Care. 2013 May;58(5):e64-5. |
| LETTER | | The authors respond to: Noninvasive mechanical ventilation and helmet after lung resection: oxygenation improvement: a small step or a large step? | | | | Ampollini L, Barbagallo M, Ziegler S, Ortu A. | Respir Care. 2013 May;58(5):e66. |
| NOT RELEVANT | | Early light reduction for preventing retinopathy of prematurity in very low birth weight infants | | | | Jorge EC, Jorge EN, El Dib RP. | Cochrane Database Syst Rev. 2013 Aug 6;2013(8):CD000122. doi: 10.1002/14651858.CD000122.pub2. |
| NOT RELEVANT | | On-road and wind-tunnel measurement of motorcycle helmet noise | | | | Kennedy J, Carley M, Walker I, Holt N. | J Acoust Soc Am. 2013 Sep;134(3):2004-10. doi: 10.1121/1.4817913. |
| NOT RELEVANT | | Ergonomic and usability ratings of helmets and head-mounted personal protective equipment in industry | | | | Godwin AA, Eger TR. | Work. 2014;47(1):23-31. doi: 10.3233/WOR-131687. |
| NOT RELEVANT | | Efficacy of passive helmet therapy for deformational plagiocephaly: report of 1050 cases | | | | Couture DE, Crantford JC, Somasundaram A, Sanger C, Argenta AE, David LR. | Neurosurg Focus. 2013 Oct;35(4):E4. doi: 10.3171/2013.8.FOCUS13258. |
| NOT RELEVANT | | Sport helmet design and virtual impact test by image-based finite element modeling | | | | Luo Y, Liang Z. | Annu Int Conf IEEE Eng Med Biol Soc. 2013;2013:7237-40. doi: 10.1109/EMBC.2013.6611228. |
| NOT RELEVANT | | Are water precautions necessary after tympanostomy tube placement? | | | | Tsao GJ, Goode RL. | Laryngoscope. 2014 Jul;124(7):1513-4. doi: 10.1002/lary.24473. Epub 2013 Dec 18. |
| LANGUAGE | | [Invasive and non-invasive ventilation: impact on nursing workload] | | | | Lucchini A, Elli S, Bambi S, Foti G, Fumagalli R. | Assist Inferm Ric. 2013 Jul-Sep;32(3):124-31. doi: 10.1702/1338.14853. |
| NOT RELEVANT | | Oxygen mask fit analysis in F-16 fighter pilots using 3D imaging | | | | Schreinemakers JR, Oudenhuijzen AJ, van Amerongen PC, Kon M. | Aviat Space Environ Med. 2013 Oct;84(10):1029-33. doi: 10.3357/asem.3611.2013. |
| CASE REPORT/SERIES | | Transesophageal echocardiography through a non-invasive ventilation helmet | | | | Pisano A, Angelone M, Iovino T, Gargiulo S, Manduca S, De Pietro A. | J Cardiothorac Vasc Anesth. 2013 Dec;27(6):e78-81. doi: 10.1053/j.jvca.2013.08.007. |
| NOT RELEVANT | | Effect of wearing a ski helmet on perception and localization of sounds | | | | Ruedl G, Kopp M, Burtscher M, Zorowka P, Weichbold V, Stephan K, Koci V, Seebacher J. | Int J Sports Med. 2014 Jul;35(8):645-50. doi: 10.1055/s-0033-1358673. Epub 2014 Jan 9. |
| NOT RELEVANT | | Motorcycle injuries as an emerging public health problem in Mwanza City, north-western Tanzania | | | | Chalya PL, Mabula JB, Ngayomela IH, Kanumba ES, Chandika AB, Giiti G, Mawala B, Balumuka DD. | Tanzan J Health Res. 2010 Oct;12(4):214-21. |
| LANGUAGE | | [Interface type helmet non-invasive mechanical ventilation] | | | | Migallón Buitrago ME, García-Velasco Sánchez-Morago S, Ramírez de Orol MA, Puyana Manrique de Lara Mdel C. | Rev Enferm. 2013 Dec;36(12):34-40. |
| NOT RELEVANT | | Helmet liner evaluation to mitigate head response from primary blast exposure | | | | Lockhart PA, Cronin DS. | Comput Methods Biomech Biomed Engin. 2015;18(6):635-45. doi: 10.1080/10255842.2013.829460. Epub 2014 Feb 24. |
| NOT RELEVANT | | Repeal of the Michigan helmet law: early clinical impacts | | | | Chapman AJ, Titus R, Ferenchick H, Davis A, Rodriguez C. | Am J Surg. 2014 Mar;207(3):352-6; discussion 355-6. doi: 10.1016/j.amjsurg.2013.12.001. Epub 2013 Dec 25. |
| NOT RELEVANT | | Three-dimensional finite element modeling of the human external ear: simulation study of the bone conduction occlusion effect | | | | Brummund MK, Sgard F, Petit Y, Laville F. | J Acoust Soc Am. 2014 Mar;135(3):1433-44. doi: 10.1121/1.4864484. |
| LANGUAGE | | [Airway devices in the intensive care unit] | | | | Dreher M, Kluge S. | Pneumologie. 2014 Jun;68(6):371-7. doi: 10.1055/s-0034-1365318. Epub 2014 Mar 25. |
| NOT RELEVANT | | Dynamic response due to behind helmet blunt trauma measured with a human head surrogate | | | | Freitas CJ, Mathis JT, Scott N, Bigger RP, Mackiewicz J. | Int J Med Sci. 2014 Mar 8;11(5):409-25. doi: 10.7150/ijms.8079. eCollection 2014. |
| NOT RELEVANT | | Prevention and management of pressure ulcers: support surfaces | | | | Moore Z, Stephen Haynes J, Callaghan R. | Br J Nurs. 2014 Mar 27-Apr 9;23(6):S36, S38-43. doi: 10.12968/bjon.2014.23.Sup6.S36. |
| NOT RELEVANT | | Spiked helmet sign: An under-recognized electrocardiogram finding in critically ill patients | | | | Agarwal A, Janz TG, Garikipati NV. | Indian J Crit Care Med. 2014 Apr;18(4):238-40. doi: 10.4103/0972-5229.130576. |
| CASE REPORT | | Respiratory failure due to upper airway obstruction in children: use of the helmet as bridge interface | | | | Racca F, Cutrera R, Robba C, Caldarelli V, Paglietti MG, De Angelis MC, Sekhon MS, Gualino J, Bella C, Passoni N, Ranieri VM. | Minerva Anestesiol. 2015 Feb;81(2):175-8. Epub 2014 Jun 11. |
| NOT RELEVANT | | A description of the severity of equestrian-related injuries (ERIs) using clinical parameters and patient-reported outcomes | | | | Papachristos A, Edwards E, Dowrick A, Gosling C. | Injury. 2014 Sep;45(9):1484-7. doi: 10.1016/j.injury.2014.04.017. Epub 2014 May 2. |
| NOT RELEVANT | | Protective effect of unilateral and bilateral ear plugs on noise-induced hearing loss: functional and morphological evaluation in animal model | | | | Kim DK, Park Y, Back SA, Kim HL, Park HE, Park KH, Yeo SW, Park SN. | Noise Health. 2014 May-Jun;16(70):149-56. doi: 10.4103/1463-1741.134915. |
| NOT RELEVANT | | Exerted pressure by an in-flight oxygen mask | | | | Schreinemakers JR, Boer C, van Amerongen PC, Kon M. | Aviat Space Environ Med. 2014 Jul;85(7):745-9. doi: 10.3357/asem.3871.2014. |
| VETERINARY | | Noninvasive continuous positive airway pressure delivered using a pediatric helmet in dogs recovering from general anesthesia | | | | Staffieri F, Crovace A, De Monte V, Centonze P, Gigante G, Grasso S. | J Vet Emerg Crit Care (San Antonio). 2014 Sep-Oct;24(5):578-85. doi: 10.1111/vec.12210. Epub 2014 Aug 14. |
| NOT RELEVANT | | Simulated effects of head movement on contact pressures between headforms and N95 filtering facepiece respirators-part 1: headform model and validation | | | | Lei Z, Ji X, Li N, Yang J, Zhuang Z, Rottach D. | Ann Occup Hyg. 2014 Nov;58(9):1175-85. doi: 10.1093/annhyg/meu051. Epub 2014 Sep 3. |
| NOT RELEVANT | | Simulated effects of head movement on contact pressures between headforms and N95 filtering facepiece respirators part 2: simulation | | | | Lei Z, Ji X, Li N, Yang J, Zhuang Z, Rottach D. | Ann Occup Hyg. 2014 Nov;58(9):1186-99. doi: 10.1093/annhyg/meu064. Epub 2014 Sep 3. |
| NOT RELEVANT | | Spacecraft occupant protection requirements: a review of the recent changes | | | | Somers JT, Gohmert DM, Brinkley JW. | Aviat Space Environ Med. 2014 Sep;85(9):940-8. doi: 10.3357/ASEM.4004.2014. |
| NOT RELEVANT | | Confined space ventilation by shipyard welders: observed use and effectiveness | | | | Pouzou JG, Warner C, Neitzel RL, Croteau GA, Yost MG, Seixas NS. | Ann Occup Hyg. 2015 Jan;59(1):116-21. doi: 10.1093/annhyg/meu070. Epub 2014 Sep 22. |
| NOT RELEVANT | | Minimisation of the explosion shock wave load onto the occupants inside the vehicle during trinitrotoluene charge blast | | | | Krzystała E, Mężyk A, Kciuk S. | Int J Inj Contr Saf Promot. 2016;23(2):170-8. doi: 10.1080/17457300.2014.966118. Epub 2014 Oct 13. |
| NOT RELEVANT | | [Comfort and noise level in infants with helmet interface] | | | | Medina A, Alvarez Fernández P, Rey Galán C, Álvarez Mendiola P, Álvarez Blanco S, Vivanco Allende A. | An Pediatr (Barc). 2015 Oct;83(4):272-6. doi: 10.1016/j.anpedi.2015.02.010. Epub 2015 Mar 29. |
| NOT RELEVANT | | An Investigation of Operational Decision Making in Situ: Incident Command in the U.K. Fire and Rescue Service | | | | Cohen-Hatton SR, Butler PC, Honey RC. | Hum Factors. 2015 Aug;57(5):793-804. doi: 10.1177/0018720815578266. Epub 2015 Mar 30. |
| NOT RELEVANT | | The five-minute prebreathe in evaluating carbon dioxide absorption in a closed-circuit rebreather: a randomized single-blind study | | | | Deng C, Pollock NW, Gant N, Hannam JA, Dooley A, Mesley P, Mitchell SJ. | Diving Hyperb Med. 2015 Mar;45(1):16-24. |
| NOT RELEVANT | | Operating theatre ventilation systems and microbial air contamination in total joint replacement surgery: results of the GISIO-ISChIA study | | | | Agodi A, Auxilia F, Barchitta M, Cristina ML, D'Alessandro D, Mura I, Nobile M, Pasquarella C; Italian Study Group of Hospital Hygiene. | J Hosp Infect. 2015 Jul;90(3):213-9. doi: 10.1016/j.jhin.2015.02.014. Epub 2015 Mar 27. |
| CASE REPORT | | NIV-Helmet in Severe Hypoxemic Acute Respiratory Failure | | | | Martins J, Nunes P, Silvestre C, Abadesso C, Loureiro H, Almeida H. | Case Rep Pediatr. 2015;2015:456715. doi: 10.1155/2015/456715. Epub 2015 Apr 27. |
| NOT RELEVANT | | Effect of mechanical optical clearing on near-infrared spectroscopy | | | | Idelson CR, Vogt WC, King-Casas B, LaConte SM, Rylander CG. | Lasers Surg Med. 2015 Aug;47(6):495-502. doi: 10.1002/lsm.22373. Epub 2015 Jun 3. |
| NOT RELEVANT | | Unusual cause of a facial pressure ulcer: the helmet securing the Sengstaken-Blakemore tube | | | | Kim SM, Ju RK, Lee JH, Jun YJ, Kim YJ. | J Wound Care. 2015 Jun;24(6 Suppl):S14-6. doi: 10.12968/jowc.2015.24.Sup6.S14. |
| LETTER | | A new strategy to deliver Helmet CPAP to critically ill patients: the possible role of ICU ventilators | | | | Grieco DL, Biancone M, Maviglia R, Antonelli M. | Minerva Anestesiol. 2015 Oct;81(10):1144-5. Epub 2015 Jul 24. |
| NOT RELEVANT | | Respiratory source control using a surgical mask: An in vitro study | | | | Patel RB, Skaria SD, Mansour MM, Smaldone GC. | J Occup Environ Hyg. 2016 Jul;13(7):569-76. doi: 10.1080/15459624.2015.1043050. |
| CASE REPORT/SERIES | | Effects of Noninvasive Positive-Pressure Ventilation with Different Interfaces in Patients with Hypoxemia after Surgery for Stanford Type A Aortic Dissection | | | | Yang Y, Sun L, Liu N, Hou X, Wang H, Jia M. | Med Sci Monit. 2015 Aug 7;21:2294-304. doi: 10.12659/MSM.893956. |
| NOT RELEVANT | | A Two-Model Approach to Investigate the Mechanisms Underlying Blast-Induced Traumatic Brain Injury | | | | Chen H, Constantini S, Chen Y. | In: Kobeissy FH, editor. Brain Neurotrauma: Molecular, Neuropsychological, and Rehabilitation Aspects. Boca Raton (FL): CRC Press/Taylor & Francis; 2015. Chapter 17. |
| NOT RELEVANT | | The hazards of off-road motor sports: Are four wheels better than two? | | | | Villegas CV, Bowman SM, Zogg CK, Scott VK, Haut ER, Stevens KA, Efron DT, Haider AH. | Injury. 2016 Jan;47(1):178-83. doi: 10.1016/j.injury.2015.08.001. Epub 2015 Aug 21. |
| NOT RELEVANT | | Water precautions following ventilation tube insertion: what information are patients given? Our Experience | | | | Ridgeon E, Lawrence R, Daniel M. | Clin Otolaryngol. 2016 Aug;41(4):412-6. doi: 10.1111/coa.12530. Epub 2016 Feb 8. |
| NOT RELEVANT | | Characterization of Tungsten Inert Gas (TIG) Welding Fume Generated by Apprentice Welders | | | | Graczyk H, Lewinski N, Zhao J, Concha-Lozano N, Riediker M. | Ann Occup Hyg. 2016 Mar;60(2):205-19. doi: 10.1093/annhyg/mev074. Epub 2015 Oct 12. |
| NOT RELEVANT | | Study of contact characteristics between a respirator and a headform | | | | Cai M, Shen S, Li H, Zhang X, Ma Y. | J Occup Environ Hyg. 2016;13(3):D50-60. doi: 10.1080/15459624.2015.1116699. |
| NOT RELEVANT | | A Head and Neck Support Device for Inducing Local Hypothermia | | | | Gladen A, Iaizzo PA, Bischof JC, Erdman AG, Divani AA. | J Med Device. 2014 Mar;8(1):0110021-110029. doi: 10.1115/1.4025448. Epub 2013 Dec 6. |
| LANGUAGE | | [Analysis of the Factors in Successful Helmet Non-invasive Positive Pressure Ventilation] | | | | Ishizaki H, Terao Y, Taniguchi M, Matsumoto S, Sakai A, Egashira S, Tsuji C, Fukusaki M, Hara T. | Masui. 2015 Oct;64(10):1023-9. |
| NOT RELEVANT | | Repeal of the Michigan helmet law: the evolving clinical impact | | | | Striker RH, Chapman AJ, Titus RA, Davis AT, Rodriguez CH. | Am J Surg. 2016 Mar;211(3):529-33. doi: 10.1016/j.amjsurg.2015.11.004. Epub 2015 Dec 20. |
| NOT RELEVANT | | Mechanical damage of tympanic membrane in relation to impulse pressure waveform - A study in chinchillas | | | | Gan RZ, Nakmali D, Ji XD, Leckness K, Yokell Z. | Hear Res. 2016 Oct;340:25-34. doi: 10.1016/j.heares.2016.01.004. Epub 2016 Jan 22. |
| NOT RELEVANT | | Water precautions for prevention of infection in children with ventilation tubes (grommets) | | | | Moualed D, Masterson L, Kumar S, Donnelly N. | Cochrane Database Syst Rev. 2016 Jan 27;(1):CD010375. doi: 10.1002/14651858.CD010375.pub2. |
| NOT RELEVANT | | Simulative investigation on head injuries of electric self-balancing scooter riders subject to ground impact | | | | Xu J, Shang S, Qi H, Yu G, Wang Y, Chen P. | Accid Anal Prev. 2016 Apr;89:128-41. doi: 10.1016/j.aap.2016.01.013. Epub 2016 Feb 8. |
| NOT RELEVANT | | Inducible Defenses with a "Twist": Daphnia barbata Abandons Bilateral Symmetry in Response to an Ancient Predator | | | | Herzog Q, Rabus M, Wolfschoon Ribeiro B, Laforsch C. | PLoS One. 2016 Feb 17;11(2):e0148556. doi: 10.1371/journal.pone.0148556. eCollection 2016. |
| NOT RELEVANT | | Real-Time Detection and Monitoring of Acute Brain Injury Utilizing Evoked Electroencephalographic Potentials | | | | Fisher JA, Huang S, Ye M, Nabili M, Wilent WB, Krauthamer V, Myers MR, Welle CG. | IEEE Trans Neural Syst Rehabil Eng. 2016 Sep;24(9):1003-1012. doi: 10.1109/TNSRE.2016.2529663. Epub 2016 Mar 1. |
| NOT RELEVANT | | Football Equipment Removal Improves Chest Compression and Ventilation Efficacy | | | | Mihalik JP, Lynall RC, Fraser MA, Decoster LC, De Maio VJ, Patel AP, Swartz EE. | Prehosp Emerg Care. 2016 Sep-Oct;20(5):578-85. doi: 10.3109/10903127.2016.1149649. Epub 2016 Mar 17. |
| NOT RELEVANT | | Effectiveness of Earplugs in Preventing Recreational Noise-Induced Hearing Loss: A Randomized Clinical Trial | | | | Ramakers GG, Kraaijenga VJ, Cattani G, van Zanten GA, Grolman W. | JAMA Otolaryngol Head Neck Surg. 2016 Jun 1;142(6):551-8. doi: 10.1001/jamaoto.2016.0225. |
| NOT RELEVANT | | Is Transducer Hygiene sufficient when Vaginal Probes are used in the Clinical Routine? | | | | Merz E. | Ultraschall Med. 2016 Apr;37(2):137-9. doi: 10.1055/s-0042-103605. Epub 2016 Apr 8. |
| REVIEW/EDITORIAL | | Unmasking a Role for Noninvasive Ventilation in Early Acute Respiratory Distress Syndrome | | | | Beitler JR, Owens RL, Malhotra A. | JAMA. 2016 Jun 14;315(22):2401-3. doi: 10.1001/jama.2016.5987. |
| REVIEW/EDITORIAL | | Non-invasive ventilation by helmet more effective than face mask in acute respiratory distress syndrome | | | | Marshall H. | Lancet Respir Med. 2016 Aug;4(8):610. doi: 10.1016/S2213-2600(16)30183-7. Epub 2016 Jun 28. |
| CASE REPORT/SERIES | | Noninvasive Positive-Pressure Ventilation in Treatment of Hypoxemia After Extubation Following Type-A Aortic Dissection | | | | Yang Y, Liu N, Sun L, Zhou Y, Yang Y, Shang W, Li X. | J Cardiothorac Vasc Anesth. 2016 Dec;30(6):1539-1544. doi: 10.1053/j.jvca.2016.03.129. Epub 2016 Mar 10. |
| LANGUAGE | | [The advances of noninvasive ventilation with helmet] | | | | Liu Q, Chen RC, Cheng Z. | Zhonghua Jie He He Hu Xi Za Zhi. 2016 Sep;39(9):723-6. doi: 10.3760/cma.j.issn.1001-0939.2016.09.014. |
| NOT RELEVANT | | Can EMS Providers Provide Appropriate Tidal Volumes in a Simulated Adult-sized Patient with a Pediatric-sized Bag-Valve-Mask? | | | | Siegler J, Kroll M, Wojcik S, Moy HP. | Prehosp Emerg Care. 2017 Jan-Feb;21(1):74-78. doi: 10.1080/10903127.2016.1227003. Epub 2016 Oct 3. |
| LETTER | | Face Mask vs Helmet for Noninvasive Ventilation | | | | Taccone P, Chiumello D. | JAMA. 2016 Oct 11;316(14):1496. doi: 10.1001/jama.2016.13852. |
| LETTER | | Face Mask vs Helmet for Noninvasive Ventilation-Reply | | | | Patel BK, Hall JB, Kress JP. | JAMA. 2016 Oct 11;316(14):1497. doi: 10.1001/jama.2016.13858. |
| REVIEW/EDITORIAL | | Noninvasive ventilation for acute respiratory distress syndrome: the importance of ventilator settings | | | | Tucci MR, Costa EL, Nakamura MA, Morais CC. | J Thorac Dis. 2016 Sep;8(9):E982-E986. doi: 10.21037/jtd.2016.09.29. |
| NOT RELEVANT | | Pediatric bicycle-related head injuries: a population-based study in a county without a helmet law | | | | Kaushik R, Krisch IM, Schroeder DR, Flick R, Nemergut ME. | Inj Epidemiol. 2015 Dec;2(1):16. doi: 10.1186/s40621-015-0048-1. Epub 2015 Jul 7. |
| REVIEW/EDITORIAL | | A new horizon for the use of non-invasive ventilation in patients with acute respiratory distress syndrome | | | | Carron M. | Ann Transl Med. 2016 Sep;4(18):348. doi: 10.21037/atm.2016.09.17. |
| REVIEW/EDITORIAL | | Non-invasive ventilation in acute respiratory distress syndrome: helmet use saves lives? | | | | Sehgal IS, Agarwal R. | Ann Transl Med. 2016 Sep;4(18):349. doi: 10.21037/atm.2016.08.09. |
| REVIEW/EDITORIAL | | Should we carry out noninvasive ventilation using a helmet in acute respiratory distress syndrome? | | | | Coudroy R, Frat JP, Thille AW. | Ann Transl Med. 2016 Sep;4(18):351. doi: 10.21037/atm.2016.08.35. |
| REVIEW/EDITORIAL | | Non-invasive ventilation in hypoxemic patients: does the interface make a difference? | | | | Longhini F, Abdalla K, Navalesi P. | Ann Transl Med. 2016 Sep;4(18):359. doi: 10.21037/atm.2016.09.20. |
| 27885969 | Crit Care. 2016 Apr 20;20(Suppl 2):94. doi: 10.1186/s13054-016-1208-6. | | Bateman RM |  |  |  |  |
| REVIEW/EDITORIAL | | Prevention and treatment of skin lesions associated with non-invasive mechanical ventilation. Recommendations of experts | | | | Raurell-Torredà M, Romero-Collado A, Rodríguez-Palma M, Farrés-Tarafa M, Martí JD, Hurtado-Pardos B, Peñarrubia-San Florencio L, Saez-Paredes P, Esquinas AM. | Enferm Intensiva. 2017 Jan-Mar;28(1):31-41. doi: 10.1016/j.enfi.2016.12.001. Epub 2017 Jan 31. |
| REVIEW/EDITORIAL | | Non-invasive ventilation improves respiratory distress in children with acute viral bronchiolitis: a systematic review | | | | Combret Y, Prieur G, LE Roux P, Médrinal C. | Minerva Anestesiol. 2017 Jun;83(6):624-637. doi: 10.23736/S0375-9393.17.11708-6. Epub 2017 Feb 13. |
| NOT RELEVANT | | Moderate blast exposure results in increased IL-6 and TNFα in peripheral blood | | | | Gill J, Motamedi V, Osier N, Dell K, Arcurio L, Carr W, Walker P, Ahlers S, Lopresti M, Yarnell A. | Brain Behav Immun. 2017 Oct;65:90-94. doi: 10.1016/j.bbi.2017.02.015. Epub 2017 Feb 21. |
| NOT RELEVANT | | The impact of helmet use on outcomes after a motorcycle crash | | | | Khor D, Inaba K, Aiolfi A, Delapena S, Benjamin E, Matsushima K, Strumwasser AM, Demetriades D. | Injury. 2017 May;48(5):1093-1097. doi: 10.1016/j.injury.2017.02.006. Epub 2017 Feb 20. |
| NOT RELEVANT | | Preventing facial pressure ulcers in patients under non-invasive mechanical ventilation: a randomised control trial | | | | Otero DP, Domínguez DV, Fernández LH, Magariño AS, González VJ, Klepzing JV, Montesinos JV. | J Wound Care. 2017 Mar 2;26(3):128-136. doi: 10.12968/jowc.2017.26.3.128. |
| NOT RELEVANT | | Validation of Laboratory Animal and Surrogate Human Models in Primary Blast Injury Studies | | | | Chandra N, Sundaramurthy A, Gupta RK. | Mil Med. 2017 Mar;182(S1):105-113. doi: 10.7205/MILMED-D-16-00144. |
| LANGUAGE | | [TWO CASES OF YOUNG CHILDREN WITH ACUTE SEVERE ASTHMA TREATED BY NONINVASIVE POSITIVE PRESSURE VENTILATION VIA A HELMET] | | | | Amimoto Y, Tamura N, Kitaura N, Hirata O, Arashin O, Wago M. | Arerugi. 2017;66(2):112-117. doi: 10.15036/arerugi.66.112. |
| NOT RELEVANT | | New setting of neurally adjusted ventilatory assist for noninvasive ventilation by facial mask: a physiologic study | | | | Longhini F, Pan C, Xie J, Cammarota G, Bruni A, Garofalo E, Yang Y, Navalesi P, Qiu H. | Crit Care. 2017 Jul 7;21(1):170. doi: 10.1186/s13054-017-1761-7. |
| CASE REPORT/SERIES | | Helmet-Delivered Respiratory Support in Neonate with Severe Facial Malformation | | | | Frassoni E, Shankar-Aguilera S, Yousef N, De Luca D. | J Paediatr Child Health. 2017 Aug;53(8):825. doi: 10.1111/jpc.13635. |
| REVIEW/EDITORIAL | | High-flow nasal oxygen therapy and noninvasive ventilation in the management of acute hypoxemic respiratory failure | | | | Frat JP, Coudroy R, Marjanovic N, Thille AW. | Ann Transl Med. 2017 Jul;5(14):297. doi: 10.21037/atm.2017.06.52. |
| CASE REPORT/SERIES | | Pneumopericardium, pneumomediastinum, and pneumorrachis complicating acute respiratory syncytial virus bronchiolitis in children | | | | Fantacci C, Ferrara P, Franceschi F, Chiaretti A. | Eur Rev Med Pharmacol Sci. 2017 Aug;21(15):3465-3468. |
| REVIEW/EDITORIAL | | Ten important articles on noninvasive ventilation in critically ill patients and insights for the future: A report of expert opinions | | | | Cortegiani A, Russotto V, Antonelli M, Azoulay E, Carlucci A, Conti G, Demoule A, Ferrer M, Hill NS, Jaber S, Navalesi P, Pelosi P, Scala R, Gregoretti C. | BMC Anesthesiol. 2017 Sep 4;17(1):122. doi: 10.1186/s12871-017-0409-0. |
| REVIEW/EDITORIAL | | Choosing the Proper Interface for Positive Airway Pressure Therapy in Subjects With Acute Respiratory Failure | | | | BaHammam AS, Singh TD, Gupta R, Pandi-Perumal SR. | Respir Care. 2018 Feb;63(2):227-237. doi: 10.4187/respcare.05787. Epub 2017 Oct 31. |
| REVIEW/EDITORIAL | | J Intensive Care Soc. 2017 Nov;18(4):326-328. doi: 10.1177/1751143717700569. Epub 2017 Apr 19. | | |  |  |  |
| NOT RELEVANT | | Grain Entrapment Pressure on the Torso: Can You Breathe while Buried in Grain? | | | | Moore KG, Jones CL. | J Agric Saf Health. 2017 Apr 26;23(2):99-107. doi: 10.13031/jash.11648. |
| NOT RELEVANT | | Congenital cavitary optic disc anomaly and Axenfeld's anomaly in Wolf-Hirschhorn syndrome: A case report and review of the literature | | | | Ali MH, Azar NF, Aakalu V, Chau FY, Abbasian J, Setabutr P, Maumenee IH. | Ophthalmic Genet. 2018 Apr;39(2):271-274. doi: 10.1080/13816810.2017.1408850. Epub 2017 Dec 4. |
| REVIEW/EDITORIAL | | Mechanical ventilation in brain injured patients: seeing the forest for the trees | | | | Bruni A, Garofalo E, Pelaia C, Longhini F, Navalesi P. | J Thorac Dis. 2017 Oct;9(10):3483-3487. doi: 10.21037/jtd.2017.08.149. |
| NOT RELEVANT | | Comparison Of The I-Gel Supraglottic And King Laryngotracheal Airways In A Simulated Tactical Environment | | | | March JA, Tassey TE, Resurreccion NB, Portela RC, Taylor SE. | Prehosp Emerg Care. 2018 May-Jun;22(3):385-389. doi: 10.1080/10903127.2017.1399183. Epub 2018 Jan 24. |
| NOT RELEVANT | | What Can We Apply to Manage Acute Exacerbation of Chronic Obstructive Pulmonary Disease with Acute Respiratory Failure? | | | | Kim DK, Lee J, Park JH, Yoo KH. | Tuberc Respir Dis (Seoul). 2018 Apr;81(2):99-105. doi: 10.4046/trd.2017.0094. Epub 2018 Jan 24. |
| NOT RELEVANT | | Airway Management in Athletes Wearing Lacrosse Equipment | | | | Bowman TG, Boergers RJ, Lininger MR. | J Athl Train. 2018 Mar;53(3):240-248. doi: 10.4085/1062-6050-4-17. Epub 2018 Feb 8. |
| VETERINARY | | Comparison of three continuous positive airway pressure (CPAP) interfaces in healthy Beagle dogs during medetomidine-propofol constant rate infusions | | | | Meira C, Joerger FB, Kutter APN, Waldmann A, Ringer SK, Böehm SH, Iff S, Mosing M. | Vet Anaesth Analg. 2018 Mar;45(2):145-157. doi: 10.1016/j.vaa.2017.11.001. Epub 2017 Dec 5. |
| NOT RELEVANT | | The variations on the aerodynamics of a world-ranked wheelchair sprinter in the key-moments of the stroke cycle: A numerical simulation analysis | | | | Forte P, Marinho DA, Morais JE, Morouço PG, Barbosa TM. | PLoS One. 2018 Feb 28;13(2):e0193658. doi: 10.1371/journal.pone.0193658. eCollection 2018. |
| NOT RELEVANT | | Patellar Instability | | | | Wolfe S, Varacallo M, Thomas JD, Carroll JJ, Kahwaji CI. | 2020 Aug 16. In: StatPearls [Internet]. Treasure Island (FL): StatPearls Publishing; 2021 Jan–. |
| NOT RELEVANT | | Delivering Chest Compressions and Ventilations With and Without Men's Lacrosse Equipment | | | | Clark MD, Davis MP, Petschauer MA, Swartz EE, Mihalik JP. | J Athl Train. 2018 Apr;53(4):416-422. doi: 10.4085/1062-6050-91-17. Epub 2018 Mar 22. |
| NOT RELEVANT | | Assessment of the Effectiveness of Combat Eyewear Protection Against Blast Overpressure | | | | Sundaramurthy A, Skotak M, Alay E, Unnikrishnan G, Mao H, Duan X, Williams ST, Harding TH, Chandra N, Reifman J. | J Biomech Eng. 2018 Jul 1;140(7). doi: 10.1115/1.4039823. |
| NOT RELEVANT | | Intubation through 2 supraglottic airway device in cervical spine immobilization: a randomized trial of residents' use of the intubating laryngeal mask airway and the intubating laryngeal tube in manikins | | | | Aleksandrowicz D, Gaszyński T. | Emergencias. 2018 Jun;30(3):186-189. |
| NOT RELEVANT | | Development of new hard hat dimensions using user-centered design approach among oil palm harvesters | | | | Mohd Shukoor NS, Mohd Tamrin SB, Guan NY, Mohd Suadi Nata DH. | Work. 2018;60(1):129-134. doi: 10.3233/WOR-182741. |
| NOT RELEVANT | | Increasing Delivery of Preventive Services to Adolescents and Young Adults: Does the Preventive Visit Help? | | | | Adams SH, Park MJ, Twietmeyer L, Brindis CD, Irwin CE Jr. | J Adolesc Health. 2018 Aug;63(2):166-171. doi: 10.1016/j.jadohealth.2018.03.013. Epub 2018 Jun 19. |
| NOT RELEVANT | | Optimal pressure comfort design for pilot helmets | | | | Xu Y, Chen LY, Zhang HB, Zhao X, Tian YS, Ding L. | Comput Methods Biomech Biomed Engin. 2018 May;21(6):437-443. doi: 10.1080/10255842.2018.1478966. Epub 2018 Jul 16. |
| NOT RELEVANT | | Repetitive Head Impacts in Football Do Not Impair Dynamic Postural Control | | | | Buckley TA, Oldham JR, Watson DJ, Murray NG, Munkasy BA, Evans KM. | Med Sci Sports Exerc. 2019 Jan;51(1):132-140. doi: 10.1249/MSS.0000000000001761. |
| LETTER | | Nasal High Flow Delivered within the Helmet: A New Noninvasive Respiratory Support | | | | Mauri T, Spinelli E, Mariani M, Guzzardella A, Del Prete C, Carlesso E, Tortolani D, Tagliabue P, Pesenti A, Grasselli G. | Am J Respir Crit Care Med. 2019 Jan 1;199(1):115-117. doi: 10.1164/rccm.201806-1124LE. |
| NOT RELEVANT | | Noninvasive approach for de novo acute hypoxemic respiratory failure: noninvasive ventilation, high-flow nasal cannula, both or none? | | | | García-de-Acilu M, Patel BK, Roca O. | Curr Opin Crit Care. 2019 Feb;25(1):54-62. doi: 10.1097/MCC.0000000000000570. |
| LETTER | | Is Helmet Noninvasive Ventilation a Protective Factor for Long-Term Outcome in Acute Respiratory Distress Syndrome? | | | | Bushra M, Esquinas AM, Seetharam K. | Crit Care Med. 2019 Feb;47(2):e164. doi: 10.1097/CCM.0000000000003458. |
| NOT RELEVANT | | Limiting factors for wearing personal protective equipment (PPE) in a health care environment evaluated in a randomised study | | | | Loibner M, Hagauer S, Schwantzer G, Berghold A, Zatloukal K. | PLoS One. 2019 Jan 22;14(1):e0210775. doi: 10.1371/journal.pone.0210775. eCollection 2019. |
| NOT RELEVANT | | Point-of-care lung ultrasound in infants with bronchiolitis in the pediatric emergency department: a prospective study | | | | Supino MC, Buonsenso D, Scateni S, Scialanga B, Mesturino MA, Bock C, Chiaretti A, Giglioni E, Reale A, Musolino AM. | Eur J Pediatr. 2019 May;178(5):623-632. doi: 10.1007/s00431-019-03335-6. Epub 2019 Feb 12. |
| NOT RELEVANT | | Effects of ventilation openings in industrial safety helmets on evaporative heat dissipation | | | | Ueno S, Sawada SI. | J Occup Health. 2019 Mar;61(2):157-164. doi: 10.1002/1348-9585.12024. Epub 2019 Jan 25. |
| REVIEW/EDITORIAL | | Patient self-inflicted lung injury: implications for acute hypoxemic respiratory failure and ARDS patients on non-invasive support | | | | Grieco DL, Menga LS, Eleuteri D, Antonelli M. | Minerva Anestesiol. 2019 Sep;85(9):1014-1023. doi: 10.23736/S0375-9393.19.13418-9. Epub 2019 Mar 12. |
| LANGUAGE | | [Boussignac continuous positive airway pressure device during inter-hospital transportation in infants aged less than three months] | | | | Manso Ruiz de la Cuesta R, Del Villar Guerra P, Molinos Norniella C, Barbadillo Izquierdo F, González García J, Medina Villanueva A, Modesto Alaport V. | An Sist Sanit Navar. 2019 Apr 25;42(1):49-54. doi: 10.23938/ASSN.0587. |
| NOT RELEVANT | | Effect of Water Precautions on Otorrhea Incidence after Pediatric Tympanostomy Tube: Randomized Controlled Trial Evidence | | | | Subtil J, Jardim A, Araujo J, Moreira C, Eça T, McMillan M, Simoes Dias S, Vera Cruz P, Voegels R, Paço J, Rosenfeld R. | Otolaryngol Head Neck Surg. 2019 Sep;161(3):514-521. doi: 10.1177/0194599819844487. Epub 2019 Apr 16. |
| NOT RELEVANT | | Association of Exercise and Swimming Goggles With Modulation of Cerebro-ocular Hemodynamics and Pressures in a Model of Spaceflight-Associated Neuro-ocular Syndrome | | | | Scott JM, Tucker WJ, Martin D, Crowell JB, Goetchius E, Ozgur O, Hamilton S, Otto C, Gonzales R, Ritter M, Newby N, DeWitt J, Stenger MB, Ploutz-Snyder R, Ploutz-Snyder L, Morgan WH, Haykowsky MJ. | JAMA Ophthalmol. 2019 Jun 1;137(6):652-659. doi: 10.1001/jamaophthalmol.2019.0459. |
| NOT RELEVANT | | Autoerotic asphyxia using a plastic bag loosely covering the head over a gas mask | | | | Idota N, Nakamura M, Tsuboi H, Ichioka H, Shintani-Ishida K, Ikegaya H. | Leg Med (Tokyo). 2019 May;38:69-72. doi: 10.1016/j.legalmed.2019.04.006. Epub 2019 Apr 15. |
| NOT RELEVANT | | Motocross versus motorcycle injury patterns: A retrospective National Trauma Databank analysis | | | | Fierro N, Inaba K, Aiolfi A, Recinos G, Benjamin E, Lam L, Strumwasser A, Demetriades D. | J Trauma Acute Care Surg. 2019 Aug;87(2):402-407. doi: 10.1097/TA.0000000000002355. |
| NOT RELEVANT | | Unhelmeted Motorcycle Riders Have Increased Injury Burden: A Need to Revisit Universal Helmet Laws | | | | Patel PB, Staley CA, Runner R, Mehta S, Schenker ML. | J Surg Res. 2019 Oct;242:177-182. doi: 10.1016/j.jss.2019.03.023. Epub 2019 May 9. |
| NOT RELEVANT | | Meta-analysis of the effects of helmet-assisted non-invasive ventilation in the treatment of acute respiratory failure | | | | Zheng X, Qu NN, Wang WP, Shu P, Shi XL, Deng H, Li SJ, Qin YB. | Eur Rev Med Pharmacol Sci. 2019 May;23(10):4382-4390. doi: 10.26355/eurrev_201905_17945. |
| REVIEW/EDITORIAL | | NIV through the helmet can be used as first-line intervention for early mild and moderate ARDS: an unproven idea thinking out of the box | | | | Antonelli M. | Crit Care. 2019 Jun 14;23(Suppl 1):146. doi: 10.1186/s13054-019-2429-2. |
| NOT RELEVANT | | Electrical impedance tomography during spontaneous breathing trials and after extubation in critically ill patients at high risk for extubation failure: a multicenter observational study | | | | Longhini F, Maugeri J, Andreoni C, Ronco C, Bruni A, Garofalo E, Pelaia C, Cavicchi C, Pintaudi S, Navalesi P. | Ann Intensive Care. 2019 Aug 13;9(1):88. doi: 10.1186/s13613-019-0565-0. |
| CASE REPORT | | Helmet CPAP as a Bridge From a Rapid Response Activation for Hypoxia Through Awake Tracheostomy in the Operating Room in a Patient With a Difficult Airway: A Case Report | | | | Nocci M, Detti E, Meneguzzi M, Scolletta S. | A A Pract. 2019 Nov 15;13(10):389-391. doi: 10.1213/XAA.0000000000001092. |
| LANGUAGE | | [Meta-analysis of clinical efficacy of Helmet non-invasive ventilation and oxygen therapy on patients with hypoxemic respiratory failure] | | | | Hong S, Tian Y, Li Y, Qiao L. | Zhonghua Wei Zhong Bing Ji Jiu Yi Xue. 2019 Sep;31(9):1118-1122. doi: 10.3760/cma.j.issn.2095-4352.2019.09.012. |
| NOT RELEVANT | | The Role of Viral Coinfection in Bronchiolitis Treated With High-Flow Nasal Cannula at Pediatric Emergency Department During 2 Consecutive Seasons: An Observational Study | | | | Ferro V, Boccuzzi E, Battaglia M, Rossi FP, Olita C, Giglioni E, Concato C, Piccioni L, Perrotta D, Reale A, Raucci U. | Pediatr Infect Dis J. 2020 Feb;39(2):102-107. doi: 10.1097/INF.0000000000002512. |
| LETTER | | High Pressure versus High Flow: What Should We Target in Acute Respiratory Failure? | | | | Thille AW, Yoshida T. | Am J Respir Crit Care Med. 2020 Feb 1;201(3):265-266. doi: 10.1164/rccm.201911-2196ED. |
| NOT RELEVANT | | Consumer-Grade Headphones for Children: Limited Effectiveness of "Level Limiters" When Used With Portable or Home Media Players | | | | Stone MA, Harrison M, Wilbraham K, Lough M. | Trends Hear. 2019 Jan-Dec;23:2331216519889232. doi: 10.1177/2331216519889232. |
| LETTER | | Reply to Spinelli and Mauri: Lung and Diaphragm Protection during Noninvasive Respiratory Support | | | | Grieco DL, Menga LS, Conti G, Maggiore SM, Antonelli M. | Am J Respir Crit Care Med. 2020 Apr 1;201(7):876-878. doi: 10.1164/rccm.201912-2321LE. |
| REVIEW/EDITORIAL | | Lung and Diaphragm Protection during Noninvasive Respiratory Support | | | | Spinelli E, Mauri T. | Am J Respir Crit Care Med. 2020 Apr 1;201(7):875-876. doi: 10.1164/rccm.201911-2240LE. |
| NOT RELEVANT | | Continuous positive airway pressure (CPAP) provision with a pediatric helmet for treatment of hypoxemic acute respiratory failure in dogs | | | | Ceccherini G, Lippi I, Citi S, Perondi F, Pamapanini M, Guidi G, Briganti A. | J Vet Emerg Crit Care (San Antonio). 2020 Jan;30(1):41-49. doi: 10.1111/vec.12920. Epub 2019 Dec 23. |
| NOT RELEVANT | | Do Double-fan Surgical Helmet Systems Result in Less Gown-particle Contamination Than Single-fan Designs? | | | | Vermeiren A, Verheyden M, Verheyden F. | Clin Orthop Relat Res. 2020 Jun;478(6):1359-1365. doi: 10.1097/CORR.0000000000001121. |
| CASE REPORT | | Re-expansion pulmonary edema in a patient with anorexia nervosa and delayed drainage of traumatic pneumothorax | | | | Marongiu I, Mauri T, Spinelli E, Rosso L, Grasselli G. | AME Case Rep. 2019 Dec 3;3:46. doi: 10.21037/acr.2019.11.01. eCollection 2019. |
| NOT RELEVANT | | Primary blast wave protection in combat helmet design: A historical comparison between present day and World War I | | | | Op 't Eynde J, Yu AW, Eckersley CP, Bass CR. | PLoS One. 2020 Feb 13;15(2):e0228802. doi: 10.1371/journal.pone.0228802. eCollection 2020. |
| LANGUAGE | | [Non-invasive ventilation with helmet in patients with respiratory failure caused by acute exacerbation of chronic obstructive pulmonary disease] | | | | Liu Q, Lu H, Shan M, Wang W, Zhu C, Chen R, Zhang Z, Lan C. | Zhonghua Wei Zhong Bing Ji Jiu Yi Xue. 2020 Jan;32(1):14-19. doi: 10.3760/cma.j.cn121430-20191219-00003. |
| NOT RELEVANT | | Role of Mask/Respirator Protection Against SARS-CoV-2 | | | | Smereka J, Ruetzler K, Szarpak L, Filipiak KJ, Jaguszewski M. | Anesth Analg. 2020 Jul;131(1):e33-e34. doi: 10.1213/ANE.0000000000004873. |
| LANGUAGE | | [Position Paper for the State of the Art Application of Respiratory Support in Patients with COVID-19 - German Respiratory Society] | | | | Pfeifer M, Ewig S, Voshaar T, Randerath W, Bauer T, Geiseler J, Dellweg D, Westhoff M, Windisch W, Schönhofer B, Kluge S, Lepper PM. | Pneumologie. 2020 Jun;74(6):337-357. doi: 10.1055/a-1157-9976. Epub 2020 Apr 22. |
| REVIEW/EDITORIAL | | Helmet CPAP to Treat Acute Hypoxemic Respiratory Failure in Patients with COVID-19: A Management Strategy Proposal | | | | Radovanovic D, Rizzi M, Pini S, Saad M, Chiumello DA, Santus P. | J Clin Med. 2020 Apr 22;9(4):1191. doi: 10.3390/jcm9041191. |
| NOT RELEVANT | | COVID-19 coronavirus: recommended personal protective equipment for the orthopaedic and trauma surgeon | | | | Hirschmann MT, Hart A, Henckel J, Sadoghi P, Seil R, Mouton C. | Knee Surg Sports Traumatol Arthrosc. 2020 Jun;28(6):1690-1698. doi: 10.1007/s00167-020-06022-4. Epub 2020 Apr 27. |
| NOT RELEVANT | | Health workers' safety during tracheostomy in COVID-19 patients: Homemade protective screen | | | | Cordier PY, De La Villeon B, Martin E, Goudard Y, Haen P. | Head Neck. 2020 Jul;42(7):1361-1362. doi: 10.1002/hed.26222. Epub 2020 Apr 29. |
| NOT RELEVANT | | Skull-base surgery during the COVID-19 pandemic: the Italian Skull Base Society recommendations | | | | Castelnuovo P, Turri-Zanoni M, Karligkiotis A, Battaglia P, Pozzi F, Locatelli D; Italian Skull Base Society Board, Italian Skull Base Society Board (Società Italiana Basicranio [SIB]), Bernucci C, Iacoangeli M, Krengli M, Marchetti M, Pareschi R, Pompucci A, Rabbiosi D. | Int Forum Allergy Rhinol. 2020 Aug;10(8):963-967. doi: 10.1002/alr.22596. Epub 2020 Jun 15. |
| NOT RELEVANT | | Helmet Modification to PPE With 3D Printing During the COVID-19 Pandemic at Duke University Medical Center: A Novel Technique | | | | Erickson MM, Richardson ES, Hernandez NM, Bobbert DW 2nd, Gall K, Fearis P. | J Arthroplasty. 2020 Jul;35(7S):S23-S27. doi: 10.1016/j.arth.2020.04.035. Epub 2020 Apr 18. |
| REVIEW/EDITORIAL | | Safety Guidelines for Sterility of Face Shields During COVID 19 Pandemic | | | | Khan MM, Parab SR. | Indian J Otolaryngol Head Neck Surg. 2020 Apr 30;73(1):1-2. doi: 10.1007/s12070-020-01865-2. Online ahead of print. |
| NOT RELEVANT | | Tracheostomy during COVID-19 pandemic-Novel approach | | | | Chow VLY, Chan JYW, Ho VWY, Pang SSY, Lee GCC, Wong MMK, Lo ASH, Lui F, Poon CCM, Wong STS. | Head Neck. 2020 Jul;42(7):1367-1373. doi: 10.1002/hed.26234. Epub 2020 May 6. |
| REVIEW/EDITORIAL | | Helmet continuous positive airway pressure and prone positioning: A proposal for an early management of COVID-19 patients | | | | Longhini F, Bruni A, Garofalo E, Navalesi P, Grasselli G, Cosentini R, Foti G, Mattei A, Ippolito M, Accurso G, Vitale F, Cortegiani A, Gregoretti C. | Pulmonology. 2020 Jul-Aug;26(4):186-191. doi: 10.1016/j.pulmoe.2020.04.014. Epub 2020 Apr 30. |
| NOT RELEVANT | | Aerosol-generating otolaryngology procedures and the need for enhanced PPE during the COVID-19 pandemic: a literature review | | | | Mick P, Murphy R. | J Otolaryngol Head Neck Surg. 2020 May 11;49(1):29. doi: 10.1186/s40463-020-00424-7. |
| NOT RELEVANT | | High-Risk Aerosol-Generating Procedures in COVID-19: Respiratory Protective Equipment Considerations | | | | Howard BE. | Otolaryngol Head Neck Surg. 2020 Jul;163(1):98-103. doi: 10.1177/0194599820927335. Epub 2020 May 12. |
| NOT RELEVANT | | A Positive-Pressure Environment Disposable Shield (PEDS) for COVID-19 Health Care Worker Protection | | | | Chien LC, Beÿ CK, Koenig KL. | Prehosp Disaster Med. 2020 Aug;35(4):434-437. doi: 10.1017/S1049023X20000643. Epub 2020 May 13. |
| NOT RELEVANT | | COVID-19 response in northwest Syria: innovation and community engagement in a complex conflict | | | | Ekzayez A, Al-Khalil M, Jasiem M, Al Saleh R, Alzoubi Z, Meagher K, Patel P. | J Public Health (Oxf). 2020 Aug 18;42(3):504-509. doi: 10.1093/pubmed/fdaa068. |
| NOT RELEVANT | | Severe acute respiratory syndrome coronavirus 2 RNA contamination of inanimate surfaces and virus viability in a health care emergency unit | | | | Colaneri M, Seminari E, Novati S, Asperges E, Biscarini S, Piralla A, Percivalle E, Cassaniti I, Baldanti F, Bruno R, Mondelli MU; COVID19 IRCCS San Matteo Pavia Task Force. | Clin Microbiol Infect. 2020 Aug;26(8):1094.e1-1094.e5. doi: 10.1016/j.cmi.2020.05.009. Epub 2020 May 22. |
| CASE REPORT | | Enhanced platelet inhibition treatment improves hypoxemia in patients with severe Covid-19 and hypercoagulability. A case control, proof of concept study | | | | Viecca M, Radovanovic D, Forleo GB, Santus P. | Pharmacol Res. 2020 Aug;158:104950. doi: 10.1016/j.phrs.2020.104950. Epub 2020 May 23. |
| NOT RELEVANT | | Guidance for otolaryngology health care workers performing aerosol generating medical procedures during the COVID-19 pandemic | | | | Lammers MJW, Lea J, Westerberg BD. | J Otolaryngol Head Neck Surg. 2020 Jun 3;49(1):36. doi: 10.1186/s40463-020-00429-2. |
| NOT RELEVANT | | How and why use the EasyBreath® surface snorkeling mask as a personal protective equipment during the COVID-19 pandemic? | | | | Thierry B, Célérier C, Simon F, Lacroix C, Khonsari RH. | Eur Ann Otorhinolaryngol Head Neck Dis. 2020 Sep;137(4):329-331. doi: 10.1016/j.anorl.2020.05.006. Epub 2020 May 23. |
| NOT RELEVANT | | COVID-19 Incidentally Detected on PET/CT During Work-up for Locally Advanced Head and Neck Cancer | | | | Mo A, Brodin NP, Tomé WA, Garg MK, Kabarriti R. | In Vivo. 2020 Jun;34(3 Suppl):1681-1684. doi: 10.21873/invivo.11961. |
| NOT RELEVANT | | The COVID-19 pandemic, personal protective equipment and respirator: A narrative review | | | | Ha JF. | Int J Clin Pract. 2020 Oct;74(10):e13578. doi: 10.1111/ijcp.13578. Epub 2020 Jun 28. |
| CASE REPORT/SERIES | | Helmet-based noninvasive ventilation for acute exacerbation of chronic obstructive pulmonary disease: A case report | | | | Park MH, Kim MJ, Kim AJ, Lee MJ, Kim JS. | World J Clin Cases. 2020 May 26;8(10):1939-1943. doi: 10.12998/wjcc.v8.i10.1939. |
| NOT RELEVANT | | Fibrodysplasia Ossificans Progressiva | | | | Akesson LS, Savarirayan R. | 2020 Jun 11. In: Adam MP, Ardinger HH, Pagon RA, Wallace SE, Bean LJH, Mirzaa G, Amemiya A, editors. GeneReviews(®) [Internet]. Seattle (WA): University of Washington, Seattle; 1993–2021. |
| NOT RELEVANT | | Aerosol containment box to the rescue: extra protection for the front line | | | | Hsu SH, Lai HY, Zabaneh F, Masud FN. | Emerg Med J. 2020 Jul;37(7):400-401. doi: 10.1136/emermed-2020-209829. Epub 2020 Jun 11. |
| NOT RELEVANT | | Salivary detection of SARS-CoV-2 (COVID-19) and implications for oral health-care providers | | | | Bajaj N, Granwehr BP, Hanna EY, Chambers MS. | Head Neck. 2020 Jul;42(7):1543-1547. doi: 10.1002/hed.26322. Epub 2020 Jun 13. |
| NOT RELEVANT | | Utilization of an Orthopedic Hood as Personal Protective Equipment for Intubation of Coronavirus Patients: a Brief Technical Report | | | | Wills TT, Zuelzer WA, Tran BW. | Geriatr Orthop Surg Rehabil. 2020 Jun 2;11:2151459320930554. doi: 10.1177/2151459320930554. eCollection 2020. |
| NOT RELEVANT | | Distanciation sociale, masques, protection oculaire: comment prévenir la transmission interpersonnelle de SARS-CoV-2 | | | | Francioli P. | Rev Med Suisse. 2020 Jun 17;16(698):1274. |
| NOT RELEVANT | | Measurement of airborne particle exposure during simulated tracheal intubation using various proposed aerosol containment devices during the COVID-19 pandemic | | | | Simpson JP, Wong DN, Verco L, Carter R, Dzidowski M, Chan PY. | Anaesthesia. 2020 Dec;75(12):1587-1595. doi: 10.1111/anae.15188. Epub 2020 Jul 9. |
| NOT RELEVANT | | Does a surgical helmet provide protection against aerosol transmitted disease? | | | | Temmesfeld MJ, Jakobsen RB, Grant P. | Acta Orthop. 2020 Oct;91(5):538-542. doi: 10.1080/17453674.2020.1771525. Epub 2020 Jun 23. |
| NOT RELEVANT | | Histopathological findings in a COVID-19 patient affected by ischemic gangrenous cholecystitis | | | | Bruni A, Garofalo E, Zuccalà V, Currò G, Torti C, Navarra G, De Sarro G, Navalesi P, Longhini F, Ammendola M. | World J Emerg Surg. 2020 Jul 2;15(1):43. doi: 10.1186/s13017-020-00320-5. |
| NOT RELEVANT | | Off-the-shelf barrier for emergency intubation in the cardiac catheterization laboratory during the coronavirus disease 2019 (COVID-19) pandemic | | | | Scheller B, Vukadinovic D, Ewen S, Mahfoud F. | Clin Res Cardiol. 2020 Dec;109(12):1507-1509. doi: 10.1007/s00392-020-01696-9. Epub 2020 Jul 4. |
| NOT RELEVANT | | Risk of SARS-CoV-2 transmission by aerosols, the rational use of masks, and protection of healthcare workers from COVID-19 | | | | Sommerstein R, Fux CA, Vuichard-Gysin D, Abbas M, Marschall J, Balmelli C, Troillet N, Harbarth S, Schlegel M, Widmer A; Swissnoso. | Antimicrob Resist Infect Control. 2020 Jul 6;9(1):100. doi: 10.1186/s13756-020-00763-0. |
| NOT RELEVANT | | Geriatric ATV and snowmobile trauma at a rural level 1 trauma center: A blow to the chest | | | | Goldwag JL, Porter ED, Wilcox AR, Martin ED, Wolffing AB, Mancini DJ, Briggs A. | Injury. 2020 Sep;51(9):2040-2045. doi: 10.1016/j.injury.2020.05.043. Epub 2020 Jun 17. |
| NOT RELEVANT | | SIR HELMET (Safety In Radiology HEalthcare Localised Metrological EnviromenT): a low-cost negative-pressure isolation barrier for shielding MRI frontline workers from COVID-19 exposure | | | | Ong SJ, Renfrew I, Anil G, Tan AP, Sia SY, Low CK, Hoon HX, Ang BWL, Quek ST. | Clin Radiol. 2020 Sep;75(9):711.e1-711.e4. doi: 10.1016/j.crad.2020.06.015. Epub 2020 Jul 1. |
| NOT RELEVANT | | Recommendations for head and neck surgical procedures during the COVID-19 pandemic | | | | Kulcsar MAV, Montenegro FLM, Santos ABO, Tavares MR, Arap SS, Kowalski LP. | Clinics (Sao Paulo). 2020;75:e2084. doi: 10.6061/clinics/2020/e2084. Epub 2020 Jul 6. |
| VETERINARY | | Evaluation of the effects of helmet continuous positive airway pressure on laryngeal size in dogs anesthetized with propofol and fentanyl using computed tomography | | | | Rondelli V, Guarracino A, Iacobellis P, Grasso S, Stripoli T, Lacitignola L, Auriemma E, Romano F, Araos JD, Staffieri F. | J Vet Emerg Crit Care (San Antonio). 2020 Sep;30(5):543-549. doi: 10.1111/vec.12977. Epub 2020 Jul 10. |
| NOT RELEVANT | | Best of colleagues, worst of times | | | | Moore Z. | J Wound Care. 2020 Jul 2;29(7):377. doi: 10.12968/jowc.2020.29.7.377. |
| NOT RELEVANT | | Navigating shifting waters: rapid response to change in the era of COVID-19 | | | | Johnson DF, Steinfort DP, Cowie B. | Intern Med J. 2020 Jul;50(7):786-790. doi: 10.1111/imj.14921. |
| NOT RELEVANT | | The Drag Crisis Phenomenon on an Elite Road Cyclist-A Preliminary Numerical Simulations Analysis in the Aero Position at Different Speeds | | | | Forte P, Morais JE, P Neiva H, Barbosa TM, Marinho DA. | Int J Environ Res Public Health. 2020 Jul 11;17(14):5003. doi: 10.3390/ijerph17145003. |
| CASE REPORT | | Axillary vein thrombosis in COVID positive patient with midline and continuous positive airway pressure Helmet | | | | Vailati D, Fusco T, Canelli A, Chiariello C, Zerla P. | J Vasc Access. 2020 Jul 15:1129729820943424. doi: 10.1177/1129729820943424. Online ahead of print. |
| NOT RELEVANT | | Effect of virtual reality-simulated exercise on sympathovagal balance | | | | Ahmed S, Safdar M, Morton C, Soave N, Patel R, Castillo K, Lalande S, Jimenez L, Mateika JH, Wessells R. | PLoS One. 2020 Jul 16;15(7):e0235792. doi: 10.1371/journal.pone.0235792. eCollection 2020. |
| REVIEW/EDITORIAL | | High-Flow, Noninvasive Ventilation and Awake (Nonintubation) Proning in Patients With Coronavirus Disease 2019 With Respiratory Failure | | | | Raoof S, Nava S, Carpati C, Hill NS. | Chest. 2020 Nov;158(5):1992-2002. doi: 10.1016/j.chest.2020.07.013. Epub 2020 Jul 15. |
| NOT RELEVANT | | Sinus and anterior skull base surgery during the COVID-19 pandemic: systematic review, synthesis and YO-IFOS position | | | | Radulesco T, Lechien JR, Sowerby LJ, Saussez S, Chiesa-Estomba C, Sargi Z, Lavigne P, Calvo-Henriquez C, Lim CM, Tangjaturonrasme N, Vatanasapt P, Dehgani-Mobaraki P, Fakhry N, Ayad T, Michel J. | Eur Arch Otorhinolaryngol. 2020 Jul 24:1-10. doi: 10.1007/s00405-020-06236-9. Online ahead of print. |
| NOT RELEVANT | | The COVID-19 pandemic and face shields | | | | Ha JF. | Br J Surg. 2020 Sep;107(10):e398. doi: 10.1002/bjs.11842. Epub 2020 Jul 31. |
| NOT RELEVANT | | Protective device to reduce aerosol dispersion in dental clinics during the COVID-19 pandemic | | | | Teichert-Filho R, Baldasso CN, Campos MM, Gomes MS. | Int Endod J. 2020 Nov;53(11):1588-1597. doi: 10.1111/iej.13373. Epub 2020 Aug 18. |
| NOT RELEVANT | | Deactivation of SARS-CoV-2 with pulsed-xenon ultraviolet light: Implications for environmental COVID-19 control | | | | Simmons SE, Carrion R, Alfson KJ, Staples HM, Jinadatha C, Jarvis WR, Sampathkumar P, Chemaly RF, Khawaja F, Povroznik M, Jackson S, Kaye KS, Rodriguez RM, Stibich MA. | Infect Control Hosp Epidemiol. 2021 Feb;42(2):127-130. doi: 10.1017/ice.2020.399. Epub 2020 Aug 3. |
| NOT RELEVANT | | 3D Printed frames to enable reuse and improve the fit of N95 and KN95 respirators | | | | McAvoy M, Bui AN, Hansen C, Plana D, Said JT, Yu Z, Yang H, Freake J, Van C, Krikorian D, Cramer A, Smith L, Jiang L, Lee KJ, Li SJ, Beller B, Short M, Yu SH, Mostaghimi A, Sorger PK, LeBoeuf NR. | medRxiv. 2020 Jul 26:2020.07.20.20151019. doi: 10.1101/2020.07.20.20151019. Preprint. |
| NOT RELEVANT | | Chest physiotherapy improves lung aeration in hypersecretive critically ill patients: a pilot randomized physiological study | | | | Longhini F, Bruni A, Garofalo E, Ronco C, Gusmano A, Cammarota G, Pasin L, Frigerio P, Chiumello D, Navalesi P. | Crit Care. 2020 Aug 3;24(1):479. doi: 10.1186/s13054-020-03198-6. |
| NOT RELEVANT | | Droplet and Aerosol Generation With Endonasal Surgery: Methods to Mitigate Risk During the COVID-19 Pandemic | | | | Dharmarajan H, Freiser ME, Sim E, Boorgu DSSK, Corcoran TE, Wang EW, Gardner PA, Snyderman CH. | Otolaryngol Head Neck Surg. 2021 Feb;164(2):285-293. doi: 10.1177/0194599820949802. Epub 2020 Aug 11. |
| NOT RELEVANT | | Recommendations for Cutaneous and Aesthetic Surgeries during COVID-19 Pandemic | | | | Mysore V, Savitha AS, Venkataram A, Inamadar AC, Sanjeev A, Byalekere Chandrashekar S, Devaraj DK, Khunger N, Reddy RR, Rashi P, Salim T, Mutalik SD, Arsiwala S, Barua S, Gupta S, Sirur S, Shah S. | J Cutan Aesthet Surg. 2020 Apr-Jun;13(2):77-94. doi: 10.4103/JCAS.JCAS_83_20. |
| NOT RELEVANT | | Minimizing endoscopist facial exposure to droplets: Optimal patient-endoscopist distance and use of a barrier device | | | | Suzuki S, Gotoda T, Ikehara H, Ichijima R, Kusano C. | J Gastroenterol Hepatol. 2021 Apr;36(4):1051-1056. doi: 10.1111/jgh.15219. Epub 2020 Aug 25. |
| REVIEW/EDITORIAL | | Average Volume-Assured Pressure Support | | | | Yarrarapu SNS, Saunders H, Sanghavi D. | 2021 Jan 5. In: StatPearls [Internet]. Treasure Island (FL): StatPearls Publishing; 2021 Jan–. |
| NOT RELEVANT | | Guidance on infection control and plume management with Laser and Energy-Based Devices taking into consideration COVID-19 | | | | Sullivan JR, Rademaker M, Goodman G, Bekhor P, Al-Niaimi F. | Australas J Dermatol. 2021 Feb;62(1):37-40. doi: 10.1111/ajd.13425. Epub 2020 Aug 19. |
| VETERINARY | | Effects of continuous positive airway pressure administered by a helmet in cats under general anaesthesia | | | | Di Bella C, Araos J, Lacitignola L, Grasso S, De Marzo L, Crovace AM, Staffieri F. | J Feline Med Surg. 2021 Apr;23(4):337-343. doi: 10.1177/1098612X20951279. Epub 2020 Aug 25. |
| CASE REPORT | | Helmet CPAP revisited in COVID-19 pneumonia: A case series | | | | Rali AS, Howard C, Miller R, Morgan CK, Mejia D, Sabo J, Herlihy JP, Devarajan SR. | Can J Respir Ther. 2020 Jul 23;56:32-34. doi: 10.29390/cjrt-2020-019. eCollection 2020. |
| REVIEW/EDITORIAL | | Helmet CPAP: how an unfamiliar respiratory tool is moving into treatment options during COVID-19 in the US | | | | Armirfarzan H, Shanahan JL, Schuman R, Leissner KB. | Ther Adv Respir Dis. 2020 Jan-Dec;14:1753466620951032. doi: 10.1177/1753466620951032. |
| NOT RELEVANT | | Positive end-expiratory pressure titration in COVID-19 acute respiratory failure: electrical impedance tomography vs. PEEP/FiO(2) tables | | | | Sella N, Zarantonello F, Andreatta G, Gagliardi V, Boscolo A, Navalesi P. | Crit Care. 2020 Sep 1;24(1):540. doi: 10.1186/s13054-020-03242-5. |
| NOT RELEVANT | | Reduction of allergic rhinitis symptoms with face mask usage during the COVID-19 pandemic | | | | Dror AA, Eisenbach N, Marshak T, Layous E, Zigron A, Shivatzki S, Morozov NG, Taiber S, Alon EE, Ronen O, Zusman E, Srouji S, Sela E. | J Allergy Clin Immunol Pract. 2020 Nov-Dec;8(10):3590-3593. doi: 10.1016/j.jaip.2020.08.035. Epub 2020 Sep 2. |
| NOT RELEVANT | | Back to the basics: Hand washing is public health 101 and it works to slow down the spread of viruses | | | | Tabish HB, Basch CH. | Infect Dis Health. 2020 Nov;25(4):319-320. doi: 10.1016/j.idh.2020.08.001. Epub 2020 Sep 3. |
| NOT RELEVANT | | Efficacy of surgical helmet systems for protection against COVID-19: a double-blinded randomised control study | | | | Schaller G, Nayar SK, Erotocritou M, Overton A, Stelzhammer T, Berber O. | Int Orthop. 2021 Jan;45(1):39-42. doi: 10.1007/s00264-020-04796-3. Epub 2020 Sep 8. |
| NOT RELEVANT | | American Neurotology Society, American Otological Society, and American Academy of Otolaryngology - Head and Neck Foundation Guide to Enhance Otologic and Neurotologic Care During the COVID-19 Pandemic | | | | Kozin ED, Remenschneider AK, Blevins NH, Jan TA, Quesnel AM, Chari DA, Kesser BW, Weinstein JE, Ahsan SF, Telischi FF, Adunka OF, Weber P, Knoll RM, Coelho DH, Anne S, Franck KH, Marchioni D, Barker FG 2nd, Carter BS, Lustig LR, Bojrab DI, Bhansali SA, Westerberg BD, Lundy L, Jackler RK, Roland JT Jr, Chandrasekhar SS, Antonelli PJ, Carey JP, Welling DB, Slattery WH 3rd, Lee DJ; members of the American Neurotology Society Council, members of the American Otological Society Council, and members of the American Academy of Otolaryngology – Head and Neck Surgery Hearing Committee and Implantable Hearing Devices Committee. | Otol Neurotol. 2020 Oct;41(9):1163-1174. doi: 10.1097/MAO.0000000000002868. |
| REVIEW/EDITORIAL | | Assessment, Diagnosis, and Treatment of Dysphagia in Patients Infected With SARS-CoV-2: A Review of the Literature and International Guidelines | | | | Vergara J, Skoretz SA, Brodsky MB, Miles A, Langmore SE, Wallace S, Seedat J, Starmer HM, Bolton L, Clavé P, Freitas SV, Bogaardt H, Matsuo K, de Souza CM, Mourão LF. | Am J Speech Lang Pathol. 2020 Nov 12;29(4):2242-2253. doi: 10.1044/2020_AJSLP-20-00163. Epub 2020 Sep 22. |
| NOT RELEVANT | | Overview of different modified full-face snorkelling masks for intraoperative protection | | | | Vicini C, Cammaroto G, Meccariello G, Iannella G, Fragale M, Cacco T, Sampieri C, Guastini L, Castello E, Parrinello G, De Vito A, Gulotta G, Visconti IC, Abita P, Pelucchi S, Bianchi G, Melegatti MN, Garulli G, Bosco F, Gennaiotti A, Berrettini S, Magnani M, Troncossi M, Peretti G. | Acta Otorhinolaryngol Ital. 2020 Oct;40(5):317-324. doi: 10.14639/0392-100X-N0841. Epub 2020 Sep 24. |
| NOT RELEVANT | | Improvising the surgical helmet system for aerosol-generating procedures in the OR: Surgeon designed 3D printed mould for augmented filtration system | | | | Shah D, Sahu D, Kini A, Bagaria V. | J Clin Orthop Trauma. 2021 Jan;12(1):27-32. doi: 10.1016/j.jcot.2020.09.030. Epub 2020 Sep 24. |
| CASE REPORT/SERIES | | A case report: resolution of Chiari I malformation after helmet therapy for deformational brachycephaly | | | | Street ME, Muzaffar AR, Tanaka T. | Childs Nerv Syst. 2020 Oct 3. doi: 10.1007/s00381-020-04906-x. Online ahead of print. |
| NOT RELEVANT | | Development and Early Implementation of a Public Communication Campaign to Help Adults to Support Children and Adolescents to Cope With Coronavirus-Related Emotions: A Community Case Study | | | | Raccanello D, Vicentini G, Rocca E, Barnaba V, Hall R, Burro R. | Front Psychol. 2020 Sep 10;11:2184. doi: 10.3389/fpsyg.2020.02184. eCollection 2020. |
| NOT RELEVANT | | Impact of COVID-19 pandemic on otolaryngology, ophthalmology and dental clinical activity and future perspectives | | | | Ralli M, Candelori F, Cambria F, Greco A, Angeletti D, Lambiase A, Campo F, Minni A, Polimeni A, de Vincentiis M. | Eur Rev Med Pharmacol Sci. 2020 Sep;24(18):9705-9711. doi: 10.26355/eurrev_202009_23062. |
| NOT RELEVANT | | Sensor orientation and other factors which increase the blast overpressure reporting errors | | | | Misistia A, Skotak M, Cardenas A, Alay E, Chandra N, Kamimori GH. | PLoS One. 2020 Oct 8;15(10):e0240262. doi: 10.1371/journal.pone.0240262. eCollection 2020. |
| NOT RELEVANT | | A study on cyclist head injuries based on an electric-bicycle to car accident reconstruction | | | | Gao W, Bai Z, Li H, Liu Y, Chou CC, Jiang B. | Traffic Inj Prev. 2020;21(8):563-568. doi: 10.1080/15389588.2020.1821882. Epub 2020 Oct 14. |
| CASE REPORT | | Awake pronation with helmet continuous positive airway pressure for COVID-19 acute respiratory distress syndrome patients outside the ICU: A case series | | | | Paternoster G, Sartini C, Pennacchio E, Lisanti F, Landoni G, Cabrini L. | Med Intensiva. 2020 Sep 6:S0210-5691(20)30273-4. doi: 10.1016/j.medin.2020.08.008. Online ahead of print. |
| NOT RELEVANT | | Custom solution for personal protective equipment (PPE) in the orthopaedic setting: retrofitting Stryker Flyte T5 PPE system | | | | Gibbons JP, Hayes J, Skerritt CJ, O'Byrne JM, Green CJ. | J Hosp Infect. 2021 Feb;108:55-63. doi: 10.1016/j.jhin.2020.10.016. Epub 2020 Oct 26. |
| NOT RELEVANT | | Modification of Stryker T5(TM) and Stryker Flyte® Personal Protection Surgical Helmets to Function as Powered Air-Purifying Respirators | | | | Sachwani-Daswani GR, Atkinson P, Haake RS, Mercer L. | Surg Innov. 2020 Nov 1:1553350620967246. doi: 10.1177/1553350620967246. Online ahead of print. |
| NOT RELEVANT | | Esophageal balloon calibration during Sigh: A physiologic, randomized, cross-over study | | | | Cammarota G, Santangelo E, Lauro G, Verdina F, Boniolo E, De Vita N, Tarquini R, Spinelli E, Garofalo E, Bruni A, Zanoni M, Messina A, Pesenti A, Corte FD, Navalesi P, Vaschetto R, Mauri T. | J Crit Care. 2021 Feb;61:125-132. doi: 10.1016/j.jcrc.2020.10.021. Epub 2020 Oct 24. |
| REVIEW/EDITORIAL | | Material and Technology: Back to the Future for the Choice of Interface for Non-Invasive Ventilation - A Concise Review | | | | Scala R, Accurso G, Ippolito M, Cortegiani A, Iozzo P, Vitale F, Guidelli L, Gregoretti C. | Respiration. 2020;99(9):800-817. doi: 10.1159/000509762. Epub 2020 Nov 18. |
| NOT RELEVANT | | Eye Protection for Patients With COVID-19 Undergoing Prolonged Prone-Position Ventilation | | | | Sun L, Hymowitz M, Pomeranz HD. | JAMA Ophthalmol. 2021 Jan 1;139(1):109-112. doi: 10.1001/jamaophthalmol.2020.4988. |
| REVIEW/EDITORIAL | | Management of critically ill patients with COVID-19: suggestions and instructions from the coordination of intensive care units of Lombardy | | | | Foti G, Giannini A, Bottino N, Castelli GP, Cecconi M, Grasselli G, Guatteri L, Latronico N, Langer T, Monti G, Muttini S, Pesenti A, Radrizzani D, Ranucci M, Russotto V, Fumagalli R; COVID-19 Lombardy ICU Network. | Minerva Anestesiol. 2020 Nov;86(11):1234-1245. doi: 10.23736/S0375-9393.20.14762-X. |
| NOT RELEVANT | | Feasibility of Prehospital Rapid Sequence Intubation in the Cabin of an AW169 Helicopter | | | | McHenry AS, Curtis L, Ter Avest E, Russell MQ, Halls AV, Mitchinson S, Griggs JE, Lyon RM; Air Ambulance Kent Surrey Sussex. | Air Med J. 2020 Nov-Dec;39(6):468-472. doi: 10.1016/j.amj.2020.08.006. Epub 2020 Sep 23. |
| NOT RELEVANT | | COVID-19 and respiratory protection for healthcare providers | | | | Sozkes S, Sozkes S. | Int J Occup Med Environ Health. 2020 Nov 24:128171. doi: 10.13075/ijomeh.1896.01666. Online ahead of print. |
| NOT RELEVANT | | Clinical outcomes of high-flow nasal cannula in COVID-19 associated postextubation respiratory failure. A single-centre case series | | | | Simioli F, Annunziata A, Langella G, Polistina GE, Martino M, Fiorentino G. | Anaesthesiol Intensive Ther. 2020;52(5):373-376. doi: 10.5114/ait.2020.101007. |
| REVIEW/EDITORIAL | | Noninvasive ventilation and high-flow nasal oxygen for acute respiratory failure: is less more? | | | | Thille AW, Coudroy R, Frat JP. | Curr Opin Crit Care. 2021 Feb 1;27(1):60-65. doi: 10.1097/MCC.0000000000000785. |
| NOT RELEVANT | | Burden of motorcyclists without helmets in a state without a universal helmet law: a propensity score analysis | | | | Jones MD, Eastes JG, Veljanoski D, Chapple KM, Bogert JN, Weinberg JA. | Trauma Surg Acute Care Open. 2020 Nov 26;5(1):e000583. doi: 10.1136/tsaco-2020-000583. eCollection 2020. |
| NOT RELEVANT | | Barrier Devices, Intubation, and Aerosol Mitigation Strategies: Personal Protective Equipment in the Time of Coronavirus Disease 2019 | | | | Fried EA, Zhou G, Shah R, Shin DW, Shah A, Katz D, Burnett GW. | Anesth Analg. 2021 Jan;132(1):38-45. doi: 10.1213/ANE.0000000000005249. Epub 2020 Sep 15. |
| NOT RELEVANT | | High flow nasal therapy versus noninvasive ventilation as initial ventilatory strategy in COPD exacerbation: a multicenter non-inferiority randomized trial | | | | Cortegiani A, Longhini F, Madotto F, Groff P, Scala R, Crimi C, Carlucci A, Bruni A, Garofalo E, Raineri SM, Tonelli R, Comellini V, Lupia E, Vetrugno L, Clini E, Giarratano A, Nava S, Navalesi P, Gregoretti C; H. F.-AECOPD study investigators. | Crit Care. 2020 Dec 14;24(1):692. doi: 10.1186/s13054-020-03409-0. |
| NOT RELEVANT | | Diagnostic and therapeutic endonasal rhinologic procedures generating aerosol during COVID-19 pandemic: a systematized review | | | | Tuli IP, Trehan S, Khandelwal K, Chamoli P, Nagendra S, Tomar A, Sharma S. | Braz J Otorhinolaryngol. 2020 Dec 13:S1808-8694(20)30229-9. doi: 10.1016/j.bjorl.2020.11.008. Online ahead of print. |
| CASE REPORT | | SARS-CoV-2 pneumonia succesfully treated with cpap and cycles of tripod position: a case report | | | | Rauseo M, Mirabella L, Caporusso RR, Cantatore LP, Perrini MP, Vetuschi P, La Bella D, Tullo L, Cinnella G. | BMC Anesthesiol. 2021 Jan 8;21(1):9. doi: 10.1186/s12871-020-01221-5. |
| NOT RELEVANT | | Bench testing of noninvasive ventilation masks with viral filters for the protection from inhalation of infectious respirable particles | | | | Dellweg D, Haidl P, Kerl J, Maurer L, Köhler D. | J Occup Environ Hyg. 2021 Mar;18(3):118-127. doi: 10.1080/15459624.2020.1862417. Epub 2021 Jan 12. |
| NOT RELEVANT | | Management of tracheostomy in COVID-19 patients: The Japanese experience | | | | Yokokawa T, Ariizumi Y, Hiramatsu M, Kato Y, Endo K, Obata K, Kawashima K, Sakata T, Hirano S, Nakashima T, Sekine T, Kiyuna A, Uemura S, Okubo K, Sugimoto T, Tateya I, Fujimoto Y, Horii A, Kimura Y, Hyodo M, Homma A. | Auris Nasus Larynx. 2021 Jun;48(3):525-529. doi: 10.1016/j.anl.2021.01.006. Epub 2021 Jan 9. |
| NOT RELEVANT | | Comparison of Fit for Sealed and Loose-Fitting Surgical Masks and N95 Filtering Facepiece Respirators | | | | Karuppasamy K, Obuchowski N. | Ann Work Expo Health. 2021 Jan 18:wxaa125. doi: 10.1093/annweh/wxaa125. Online ahead of print. |
| REVIEW/EDITORIAL | | Noninvasive ventilation and high-flow oxygen therapy for severe community-acquired pneumonia | | | | Cutuli SL, Grieco DL, Menga LS, De Pascale G, Antonelli M. | Curr Opin Infect Dis. 2021 Apr 1;34(2):142-150. doi: 10.1097/QCO.0000000000000715. |
| NOT RELEVANT | | Prevention of Blast-induced Auditory Injury Using 3D Printed Helmet and Hearing Protection Device - A Preliminary Study on Biomechanical Modeling and Animal | | | | Jiang S, Gannon AN, Smith KD, Brown M, Liang J, Gan RZ. | Mil Med. 2021 Jan 25;186(Suppl 1):537-545. doi: 10.1093/milmed/usaa317. |
| NOT RELEVANT | | Optimizing Helmet Pad Placement Using Computational Predicted Injury Pattern to Reduce Mild Traumatic Brain Injury | | | | Tan XG, Matic P. | Mil Med. 2021 Jan 25;186(Suppl 1):592-600. doi: 10.1093/milmed/usaa240. |
| CASE REPORT | | Helmet mask and tocilizumab for a patient with hemophagocytic lymphohistiocytosis syndrome and COVID-19: a case report | | | | Eroglu A, Kartal S, Saral OB. | Braz J Anesthesiol. 2021 Jan-Feb;71(1):79-83. doi: 10.1016/j.bjane.2020.10.009. Epub 2020 Dec 26. |
| NOT RELEVANT | | Quantitative Assessment of Viral Dispersion Associated with Respiratory Support Devices in a Simulated Critical Care Environment | | | | Avari H, Hiebert RJ, Ryzynski AA, Levy A, Nardi J, Kanji-Jaffer H, Kiiza P, Pinto R, Plenderleith SW, Fowler RA, Mbareche H, Mubareka S. | Am J Respir Crit Care Med. 2021 Feb 3. doi: 10.1164/rccm.202008-3070OC. Online ahead of print. |
| NOT RELEVANT | | A novel box for aerosol and droplet guarding and evacuation in respiratory infection (BADGER) for COVID-19 and future outbreaks | | | | Le HD, Novak GA, Janek KC, Wang J, Huynh KN, Myer C, Weinstein A, Oberstar EL, Rasmussen J, Bertram TH. | Sci Rep. 2021 Feb 4;11(1):3179. doi: 10.1038/s41598-021-82675-6. |
| NOT RELEVANT | | Effects of Varying Levels of Inspiratory Assistance with Pressure Support Ventilation and Neurally Adjusted Ventilatory Assist on Driving Pressure in Patients Recovering from Hypoxemic Respiratory Failure | | | | Cammarota G, Verdina F, De Vita N, Boniolo E, Tarquini R, Messina A, Zanoni M, Navalesi P, Vetrugno L, Bignami E, Corte FD, De Robertis E, Santangelo E, Vaschetto R. | J Clin Monit Comput. 2021 Feb 9:1-9. doi: 10.1007/s10877-021-00668-2. Online ahead of print. |
| NOT RELEVANT | | Comparative Performance Testing of Respirator versus Surgical Mask Using a Water Droplet Spray Model | | | | Scheepers PTJ, Wertheim HFL, van Dael M, Anzion R, Holterman HJ, Teerenstra S, de Groot M, Voss A, Hopman J. | Int J Environ Res Public Health. 2021 Feb 8;18(4):1599. doi: 10.3390/ijerph18041599. |
| REVIEW/EDITORIAL | | Use of Helmet CPAP in COVID-19 - A practical review | | | | Amirfarzan H, Cereda M, Gaulton TG, Leissner KB, Cortegiani A, Schumann R, Gregoretti C. | Pulmonology. 2021 Feb 1:S2531-0437(21)00040-4. doi: 10.1016/j.pulmoe.2021.01.008. Online ahead of print. |
| NOT RELEVANT | | The use of oxygen hoods in patients failing on conventional high-flow oxygen delivery systems, the effects on oxygenation, mechanical ventilation and mortality rates in hypoxic patients with COVID-19. A Prospective Controlled Cohort Study | | | | Dayya D, O'Neill OJ, Feiertag TD, Tuazon-Boer R, Sullivan J, Perez L, Gurash S, Eaton M, Bodley T, Marker J, Smykowski E, Hall T. | Respir Med. 2021 Apr;179:106312. doi: 10.1016/j.rmed.2021.106312. Epub 2021 Feb 12. |
| CASE REPORT/SERIES | | Oxygen therapy via a noninvasive helmet: A COVID-19 novelty with potential post-pandemic uses | | | | Harrison MF, Villar D, Yarrarapu SNS, Guru P, Mallea J, Torp K, Bechtle P, Lee A, Franco PM, Sanghavi DK. | Respir Med Case Rep. 2021;32:101369. doi: 10.1016/j.rmcr.2021.101369. Epub 2021 Feb 23. |
| NOT RELEVANT | | Medical face masks offer self-protection against aerosols: An evaluation using a practical in vitro approach on a dummy head | | | | Sterr CM, Nickel IL, Stranzinger C, Nonnenmacher-Winter CI, Günther F. | PLoS One. 2021 Mar 3;16(3):e0248099. doi: 10.1371/journal.pone.0248099. eCollection 2021. |
| NOT RELEVANT | | Analysis of HIC and Hydrostatic Pressure in the Human Head during NOCSAE Tests of American Football Helmets | | | | Dymek M, Ptak M, Ratajczak M, Fernandes FAO, Kwiatkowski A, Wilhelm J. | Brain Sci. 2021 Feb 25;11(3):287. doi: 10.3390/brainsci11030287. |
| NOT RELEVANT | | Experimental Efficacy of the Face Shield and the Mask against Emitted and Potentially Received Particles | | | | Wendling JM, Fabacher T, Pébaÿ PP, Cosperec I, Rochoy M. | Int J Environ Res Public Health. 2021 Feb 17;18(4):1942. doi: 10.3390/ijerph18041942. |
| NOT RELEVANT | | The effectiveness of different types of motorcycle helmets - A scoping review | | | | Tabary M, Ahmadi S, Amirzade-Iranaq MH, Shojaei M, Sohrabi Asl M, Ghodsi Z, Azarhomayoun A, Ansari-Moghaddam A, Atlasi R, Araghi F, Shafieian M, Heydari ST, Sharif-Alhoseini M, O'Reilly G, Rahimi-Movaghar V. | Accid Anal Prev. 2021 May;154:106065. doi: 10.1016/j.aap.2021.106065. Epub 2021 Mar 6. |
| NOT RELEVANT | | Highly Sensitive Porous PDMS-Based Capacitive Pressure Sensors Fabricated on Fabric Platform for Wearable Applications | | | | Masihi S, Panahi M, Maddipatla D, Hanson AJ, Bose AK, Hajian S, Palaniappan V, Narakathu BB, Bazuin BJ, Atashbar MZ. | ACS Sens. 2021 Mar 26;6(3):938-949. doi: 10.1021/acssensors.0c02122. Epub 2021 Mar 17. |
| NOT RELEVANT | | Thermal and humid environment improvement of the protective clothing for medical use with a portable cooling device: Analysis of air supply parameters | | | | Su X, Tian S, Li H, Zhang X, Shao X, Gao J, Ye H. | Energy Build. 2021 Jun 1;240:110909. doi: 10.1016/j.enbuild.2021.110909. Epub 2021 Mar 15. |
| NOT RELEVANT | | Simulation of a vacuum helmet to contain pathogen-bearing droplets in dental and otolaryngologic outpatient interventions | | | | Jia D, Lee Baker J, Rameau A, Esmaily M. | Phys Fluids (1994). 2021 Jan 1;33(1):013307. doi: 10.1063/5.0036749. Epub 2021 Jan 12. |
| REVIEW/EDITORIAL | | Respiratory Support During the COVID-19 Pandemic: Is It Time to Consider Using a Helmet? | | | | Munshi L, Hall JB. | JAMA. 2021 Mar 25. doi: 10.1001/jama.2021.4975. Online ahead of print. |
| NOT RELEVANT | | The Cupola: an additional layer of protection for providers working in the oropharyngeal region | | | | Villa A, Grenon M. | BMC Res Notes. 2021 Mar 25;14(1):115. doi: 10.1186/s13104-021-05524-9. |
| NOT RELEVANT | | Comfort rules for face masks among healthcare workers during COVID-19 spread | | | | Maniaci A, Ferlito S, Bubbico L, Ledda C, Rapisarda V, Iannella G, La Mantia I, Grillo C, Vicini C, Privitera E, Coco S, Cammaroto G, Lechien JR, Magliulo G, Pace A, Meccariello G, Cocuzza S. | Ann Ig. 2021 Apr 2. doi: 10.7416/ai.2021.2439. Online ahead of print. |
| NOT RELEVANT | | The roles of noninvasive mechanical ventilation with helmet in patients with acute respiratory failure: A systematic review and meta-analysis | | | | Hong S, Wang H, Tian Y, Qiao L. | PLoS One. 2021 Apr 15;16(4):e0250063. doi: 10.1371/journal.pone.0250063. eCollection 2021. |
| NOT RELEVANT | | Beware of the Bicycle! An increase in paediatric bicycle related injuries during the COVID-19 period in Western Australia | | | | van Oudtshoorn S, Chiu KYC, Khosa J. | ANZ J Surg. 2021 Apr 27. doi: 10.1111/ans.16918. Online ahead of print. |
