# Supplementary material for: The use of head helmets to deliver noninvasive ventilatory support: a comprehensive review of technical aspects and clinical findings
Source: Crit Care. 2021 Sep 8;25:327. doi: 10.1186/s13054-021-03746-8 (PMC8424168; doi:10.1186/s13054-021-03746-8)
Supplement: Supplementary file 2 — Additional file 2. Color image of helmet in a clinical scenario. [file 13054_2021_3746_MOESM2_ESM.docx]

**Head Helmets to deliver non-invasive ventilation: a review of technical and clinical aspects**

Andrea Coppadoro^1^ M.D., Elisabetta Zago^1,2^ M.D., Fabio Pavan^1,2^ M.D., Giuseppe Foti^1,2^ M.D., Giacomo Bellani^1,2^ M.D. Ph.D.

^1^ASST Monza, San Gerardo Hospital, Monza, Italy

^2^ Department of Medicine and Surgery, University of Milan-Bicocca, Monza, Italy

Additional File 2


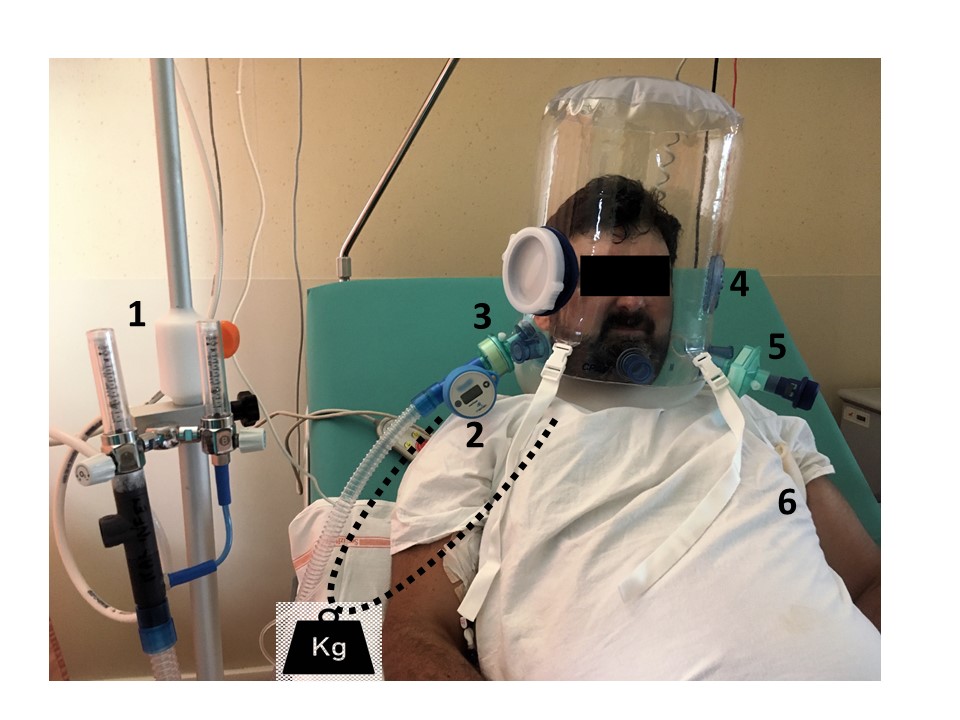


Figure e1. A typical patient undergoing helmet CPAP for COVID-19 acute respiratory failure treatment outside the ICU. A Venturi system (1) is used to generate a 60L/min flow with an FiO2 of 50%, as measured by the FiO2 meter (2). A HME filter (3) is set before helmet to reduce noise; the helmet is equipped with an anti-suffocation valve (4). An optional HEPA filter (5) is set before the PEEP valve to minimize environmental contamination. The helmet is secured by two armpit braces (6); for prolonged therapy, braces should be passed above the arms (dotted line) and connected to counterwheighs (black artwork).
